# Communication and Health in Chronic Childhood Undernutrition: Explainable Ensemble Learning to Identify Dietary Predictors of Infant Feeding Practices and Maternal Supplementation in Ecuador

**DOI:** 10.3390/nu18142232

**Published:** 2026-07-09

**Authors:** Karen Cáceres-Benítez, Ana Marcillo-Vera, Diego Almeida-Galárraga, Nathaly Orozco Garzón, Henry Carvajal Mora, Edgar Eduardo Benitez Olivo, Andrés Tirado-Espín

**Affiliations:** 1 School of Science Biological and Engineering, Yachay Tech University, Urcuquí 100119, Ecuador; karen.caceres@yachaytech.edu.ec (K.C.-B.); dalmeida@yachaytech.edu.ec (D.A.-G.); 2 School of Mathematical and Computational Sciences, Yachay Tech University, Hacienda San José s/n, Urcuquí 100119, Ecuador; ana.marcillo@yachaytech.edu.ec (A.M.-V.); ctirado@yachaytech.edu.ec (A.T.-E.); 3 ETEL Research Group, Faculty of Engineering and Applied Sciences, Networking and Telecommunications Engineering, Universidad de Las Américas (UDLA), Quito 170503, Ecuador; 4 Colegio de Ciencias e Ingenierías “El Politécnico”, Universidad San Francisco de Quito USFQ, Diego de Robles S/N, Quito 170157, Ecuador; hcarvajal@usfq.edu.ec; 5 Department of Communications, School of Electrical and Computer Engineering, University of Campinas (UNICAMP), Campinas 138083-852, SP, Brazil; ebenitez@unicamp.br

**Keywords:** chronic undernutrition, stunting, infant feeding, maternal supplementation, ENDI, explainable machine learning, SHAP, Ecuador

## Abstract

**Background/Objectives:** Chronic undernutrition in the first 1000 days continues to be a significant public health concern in Ecuador. This study examined associations between infant feeding practices, maternal supplementation, socioeconomic factors, and stunting in the first two years of life using explainable machine-learning methods. **Methods:** The analysis was based on 8613 eligible children aged 0–23 months from the Ecuadorian National Child Malnutrition Survey (Encuesta Nacional sobre Desnutrición Infantil, ENDI) 2023–2024, of whom 8344 had non-missing stunting status and were included in the final modelling sample. **Results:** Among the children included in the modelling sample, stunting was observed in 18.7%. Maternal iron and folic-acid supplementation were associated with lower stunting prevalence, while breastfeeding and complementary feeding indicators showed weak linear associations but contributed to model predictions in explainability analyses. SHapley Additive exPlanations (SHAP) additionally highlighted geographic and socioeconomic variation, with province, area of residence, and socioeconomic indicators ranking among the features contributing to model prediction. Predictive performance was modest to moderate across models, with area under the receiver operating characteristic curve (AUC) values ranging approximately from 0.62 to 0.66 across the main model specifications. Model performance remained stronger for the majority non-stunted class than for the minority stunted class, highlighting the challenge of detecting stunted children in an imbalanced analytic ENDI sample. **Conclusions:** Overall, the results suggest that stunting in the analytic sample was represented by multidimensional predictive patterns involving prenatal, nutritional, socioeconomic, and geographic factors, and illustrate the potential value of explainable machine learning for identifying multidimensional risk patterns in child nutrition research.

## 1. Introduction

Malnutrition, which refers to deficiencies, excesses or imbalances in a person’s intake of energy and/or nutrients [1], is one of the biggest barriers to achieving the Sustainable Development Goals. One of its most critical forms in early childhood is chronic undernutrition, operationalized in this study as stunting and commonly measured as a height-for-age z-score below −2 standard deviations from the WHO Child Growth Standards median [2]. According to UNICEF, around 148.1 million children under five years old worldwide were affected by stunting in 2022, representing 22.3% of all children in this age group [3,4].

Stunting reflects a long-term lack of linear growth associated with poor nutrition, infections, and inadequate care during the early stages of life [5,6,7]. It is closely linked to worse cognitive development, lower school performance, lower adult earnings, and a higher risk of noncommunicable diseases [7,8,9,10]. Although Latin America has seen overall decreases in undernutrition, the region still has significant inequalities within countries. Some countries, like Ecuador, continue to report stunting rates similar to those found in high-burden low-income areas [3,11].

In Ecuador, chronic undernutrition in early childhood is a major public health issue. National estimates from ENSANUT 2018 show that stunting in children under two years increased from 21.9% in 2006 to 27.2% in 2018. In some Andean and coastal provinces, the rates exceeded 39% [11]. Despite being an upper-middle-income country that experienced steady economic growth and rising social expenditure throughout the decade, Ecuador has failed to register proportional improvements in stunting indicators [11,12].

The first survey known as ENDI was introduced to provide quality national data about the nutritional status, feeding habits, and development of children under five years of age, especially during their first 1000 days of life [13]. This annually conducted survey, covering approximately 20,000 households and released as openly accessible data, has made it possible to investigate factors associated with malnutrition in Ecuador [13,14].

The first 1000 days, from conception until the second year, represent a key developmental period during which nutrition is closely related to brain development, immune function, and metabolic processes. Over 80% of brain growth occurs in this time frame, and inadequate nutrient intake, limited infection protection, and insufficient care during this period are associated with long-term physical and functional consequences [7,11]. Longitudinal studies show that stunting in early childhood is associated with lower educational attainment, poorer cognitive test performance, lower adult earnings, and a greater risk of obesity, type 2 diabetes, and cardiovascular disease later in life [7,15].

In Ecuador, the figures indicate that all types of malnutrition, including chronic undernutrition, are associated with an economic burden equivalent to 4.3% of the country’s gross domestic product through low productivity, higher health costs, and lower educational levels [11]. The combination of these factors shows that it is important to identify early-life nutritional leverage points.

Feeding of infants and young children (IYCF) and the nutritional status of mothers during pregnancy are fundamental to understanding linear growth during the first 1000 days. The World Health Organization (WHO) and the United Nations Children’s Fund (UNICEF) emphasize a fundamental group of infant and young child feeding indicators, which include early initiation of breastfeeding, exclusive breastfeeding for the first six months, breastfeeding continuation, dietary diversity, and adequate meal frequency [16,17]. Current evidence from low- and middle-income countries (LMICs) indicates that diet diversification and proper complementary feeding practices are positively associated with height-for-age, with lower rates of stunting among children in the 6–23-month age group [18,19,20].

The micronutrient status of pregnant women is also known to play a role in fetal development. Recent research suggests that adherence to iron-folic acid supplementation and, in certain situations, the use of multiple micronutrient supplementation during pregnancy are associated with improved outcomes, including a reduced risk of low birthweight and a lower likelihood of childhood stunting [15,21]. Likewise, iron and multimicronutrient supplementation in infants has been shown to improve hematologic outcomes and may support growth in high-risk groups; however, associations with child growth outcomes, including height-for-age, weight-for-age, and stunting-related anthropometric indicators, remain variable across settings [22,23,24]. In Ecuador, chronic undernutrition is associated with poor breastfeeding practices, low dietary diversity, and a lack of micronutrient supplementation for mothers and infants; however, the strength of these associations remains unclear [11,13,14].

Historically, classical epidemiologic studies that assessed stunting in LMICs have used linear or logistic regression models to examine the association between exposures, such as breastfeeding duration or dietary diversity scores, and height-for-age z-scores. Even though this approach has played an important role in identifying baseline associations and informing global guidelines, there is a limitation to this method because it cannot fully account for non-linear dose-response relationships, thresholds, and multivariable interactions [7,25]. Indeed, practices related to infant feeding and maternal nutrition tend to cluster, in that mothers who breastfeed their infants early may be more likely to follow supplementary feeding guidelines and offer diverse diets to their infants. Each practice is further shaped by social, economic, geographical, and health system influences, which means that analyses that isolate individual exposures may underestimate the combined predictive pattern of these exposures in relation to undernutrition and fail to leverage the multidimensional nature of comprehensive datasets like ENDI.

In addition to this, there has also been rapid advancement in the use of machine learning (ML) to predict child malnutrition from population-based survey data [26,27]. Recent results from meta-analyses of Demographic and Health Surveys (DHS) data indicate that ML models, typically random forest and gradient boosting methods, can predict child malnutrition with moderate to good accuracy (pooled accuracy ≈69% for stunting) [26].

Specifically, individual studies conducted in Africa and Asia reveal that models like random forest, XGBoost, and stacking models have been found to outperform conventional regression techniques in predicting stunting and wasting and drawing attention to complex associated factors such as poverty and environmental conditions [28,29,30,31]. However, most such approaches employ DHS module-based generic algorithms that target the under-five rather than the under-two category, and fail to leverage IYCF and maternal supplementation-related indicators as principal independent variables [25,26]. Despite the presence of microdata on ENDI [11,13,14], there is insufficient evidence in Latin America, specifically in Ecuador, about the application of sophisticated ML models in identifying predictive patterns involving infant feeding practices, maternal supplementation, and socioeconomic factors in relation to chronic undernutrition.

The issue here is that some very effective ML models are “black boxes,” thus reducing the utility of these models for program design and policy discussion related to nutrition. Methods such as those used in explainable artificial intelligence (XAI) have arisen recently and proven to be quite effective at breaking down complex ML models to the level of each predictor’s contribution to the output. These methods include Shapley Additive Explanations (SHAP), which allow researchers to examine how each predictor contributes to both individual-level and overall model predictions. Practical guides on how to apply SHAP to tree-based models and logistic regressions have been published, alongside real-world examples from clinical nutrition and malnutrition risk prediction [26,32]. On the other hand, the use of explainable ensemble learning to identify predictive patterns involving such practices in chronic undernutrition among infants and young children in Ecuador has not been extensively explored.

### 1.1. Motivation and Contribution

Given this information, the key issue addressed in this research is the high prevalence of chronic undernutrition among children aged two years or less in Ecuador. Although comprehensive data exist on the feeding practices of infants and their mothers in Ecuador, this information has yet to be used to assess how specific breastfeeding practices and supplementary feeding practices interact as predictors of stunting among young children. This study aims to employ ENDI data and explainable ensemble learning to address this challenge.

### 1.2. Research Objectives

The general goal here is to understand and explain the associations between diet, maternal nutrition factors, and chronic undernutrition in children less than 24 months old in Ecuador using explainable ensemble modeling based on ENDI data. In particular, the study aims to: (i) measure the relationship between infant and young child feeding practices and stunting during the first two years of life; (ii) estimate the association between maternal iron-folic acid supplementation during pregnancy, micronutrient supplementation for infants, and the likelihood of stunting beyond feeding practices; and (iii) develop an interpretable order of importance and response profiles of important nutritional factors.

### 1.3. Research Questions

Guided by these aims, the primary research question is: How are infant feeding practices and maternal nutritional supplementation associated with chronic undernutrition among children under two years of age in Ecuador? Two secondary questions are: (a) Which specific breastfeeding and complementary feeding indicators emerge as the strongest predictors associated with chronic undernutrition? and (b) To what extent are maternal and infant micronutrient supplementation practices associated with stunting, once feeding practices and basic sociodemographic conditions are taken into account?

### 1.4. Hypotheses

Based on the biological and epidemiological background, the study advances three hypotheses. First (H1), children who meet recommended feeding practices—early initiation of breastfeeding, exclusive breastfeeding during the first six months, and adequate dietary diversity in the complementary feeding period—will exhibit a lower probability of stunting than children with suboptimal practices. Second (H2), maternal iron–folic acid intake during pregnancy and infant micronutrient supplementation will be independently associated with lower chronic undernutrition probability, even after adjusting for infant feeding practices and key household and contextual characteristics. Third (H3), explainable ensemble learning models will capture meaningful nonlinearities and interactions among feeding and supplementation behaviours—such as interaction patterns between breastfeeding and dietary diversity—that are not adequately represented by conventional linear models, thereby offering more nuanced and policy-relevant insights into early-life dietary patterns associated with stunting in Ecuador.

In order to provide the context in which this study stands within the larger scientific body of literature and to highlight the methodological and conceptual gap that this study addresses, it becomes necessary to conduct a literature review on factors associated with stunting through diet and the use of machine learning in early childhood nutrition. The literature shows various studies that have investigated the relationship between breastfeeding practices, dietary diversity, maternal factors, micronutrient supplementation, and child outcomes; and there have also been separate lines of studies investigating the use of machine learning to predict undernutrition. However, only very few studies incorporate IYCF indicators, maternal micronutrient factors, and ensemble learning techniques. More importantly, there are no previous studies using these methodologies to analyze the ENDI datasets of Ecuador for children under 24 months of age. Table 1 provides a condensed overview of the most important literature pertinent to the present study, emphasizing the methodological approach and the main gap addressed by this work.

According to the study conducted by [33], factors associated with stunting were examined through the analysis of 395 mother-child dyads in Ethiopia by applying Bayesian logistic regression models and latent class analysis. Prevalence of stunting was quite high (47.3%), while three classes were identified. Lower stunting prevalence was associated with maternal knowledge on nutrition, dietary diversity, and spacing between births of at least 2 years. Food insecurity, poor water, sanitation, and hygiene (WASH) practices, and absence of antenatal visits were associated with higher stunting prevalence. Despite its analytical rigor, this study used no machine learning methods or World Health Organization (WHO)-established indicators for infant and young child feeding (IYCF) or anemia biomarkers.

The study by [34] employed predictive modeling and externally validated a LASSO nomogram for the prediction of stunting in more than 9500 children in China. Significant predictors included feeding practices, parental education level, and caregiver–child contact time. Although the model performed well in terms of both calibration and discrimination, comparability with the present study is limited because the study incorporated developmental outcomes measured using the Ages & Stages Questionnaires®, Third Edition (ASQ-3), rather than focusing exclusively on stunting defined by height-for-age z-score (HAZ).

However, the ML modeling efforts in [35] utilized a hypothetical dataset inspired by UNICEF, which used machine learning techniques such as XGBoost, KMeans, PCA, and reinforcement learning to identify nutritional deficiencies in children during their early years. While the results were promising, the data were not authentic, did not include anthropometric z-scores, and did not consider IYCF practices.

The cross-sectional design study by [36] analyzed the association between UPF consumption and child growth patterns amongst rural Ecuadorian children. The findings indicate that UPF consumption was associated with lower HAZ values, increased odds of being stunted, and faster bone maturity. While such findings suggest nutritional concerns related to UPF, it must be noted that due to limited data, generalization is difficult.

The study conducted in [37] based on ENSANUT data evaluated the relationship between food and water insecurity in connection with morbidity and stunting in over 20,000 children in Ecuador. Both individual and combined factors were associated with diarrhea and respiratory infection, while no association with stunting was observed. While the research offered significant information concerning the link between WASH and nutrition, it did not have dietary and IYCF data.

In the multilevel analysis by [38], individual, household, and community factors associated with the prevalence of stunting using data from MICS 2018 in Lesotho were examined. Poor maternal anthropometry, low birth weight, infections, poverty, and low educational attainment were among the significant predictors. Even though this paper was structural in nature, it lacked IYCF behaviors and micronutrients and ML techniques were not utilized.

Various factors associated with stunting were revealed in the case-control study by [39], such as poor breastfeeding practices, malnutrition related to improper complementary feeding, food insecurity, and low maternal dietary diversity. Nevertheless, it was based on inconsistent and unstandardized indicators concerning diet and did not utilize predictive modeling.

The systematic review described in [40] examined associations between micronutrient supplements during pregnancy and pregnancy or child outcomes across 72 randomized clinical trials. MMN supplementation was associated with lower LBW, SGA, stillbirth, and childhood diarrhea prevalence, while iron-folic acid supplementation was associated with lower anemia prevalence in mothers. The information gained from this study is useful to assess prenatal associated factors but insufficient to determine associations with IYCF and early childhood stunting.

In the analysis performed by [41], complementary feeding practices in Uganda were assessed using DHS data. Low MDD prevalence, appropriate MMF consumption, illness, vaccination, and wealth were the predictors identified. The study is important for assessing feeding behavior but not anthropometric outcomes, hence limiting its use in stunting research.

Lastly, [42] examined the association between relative deprivation and child growth outcomes in Ecuador based on LSMS data. Relative deprivation was a strong predictor of reduced height-for-age z-scores, with longer breastfeeding duration associated with weaker negative associations. While pertinent to socioeconomic predictors, the study lacked IYCF indicators, morbidity data, ENDI information, and machine learning models.

## 2. Methodology

**Figure 1:**
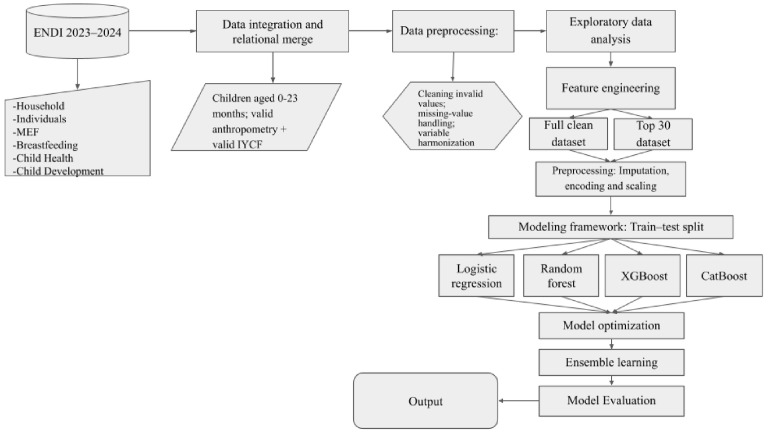
Summary of the methodological workflow used in this study.

In order to give a brief idea about the process of analysis undertaken, the major methodology steps have been depicted through a systematic flowchart. The process has been outlined in Figure 1, starting from data collection, sampling, modeling, validation, and interpretability, which help in building the predictive model for early childhood stunting in Ecuador.

### 2.1. Data Source: ENDI Database

**Figure 2:**
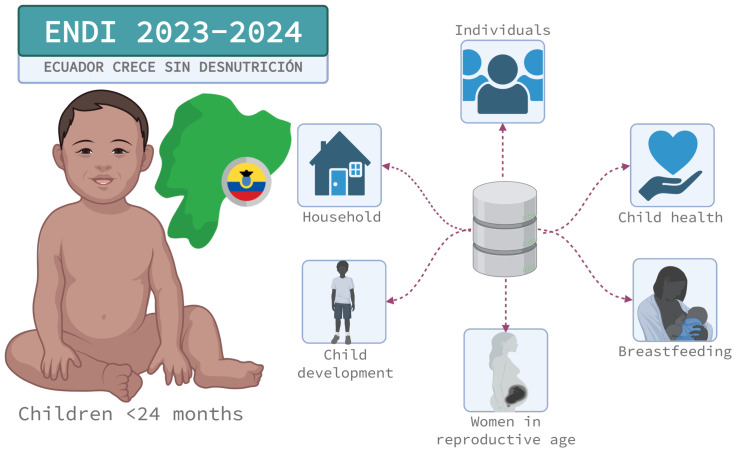
Structure of the ENDI relational databases, organized into six domains: household, individuals, women of reproductive age, breastfeeding, child health, and child development. Created in BioRender. Cáceres, K. (2026) https://BioRender.com/dzm8ub1.

The data used in this study come from the ENDI survey undertaken in Ecuador in 2023–2024. The ENDI is a nationally representative survey designed to measure the nutritional status of children and to evaluate the Ecuadorian national program Ecuador Crece Sin Desnutrición. Data collection in ENDI involves children younger than five years old and covers anthropometrics, infant and maternal nutrition, household characteristics, and early childhood development. The database is composed of six relational datasets at the household level, individuals, mothers of reproductive age (MEF), infant feeding practices, child health, and child development (see Figure 2). In this paper, we use the dataset that covers infants up to 24 months old with valid anthropometric and infant feeding variables.

According to the official ENDI methodological documentation, the 2023–2024 survey was conducted between July 2023 and August 2024 and included 22,250 investigated households, of which 20,110 were classified as effective interviews, corresponding to 90.4% effective household coverage. Documented non-effective categories included refusals (0.7%), no one at home (0.8%), temporarily occupied or vacant dwellings, and households without children under five years of age (4.9%). The ENDI also implemented quality-control procedures during data collection and processing, including standardized data-collection instruments, mobile and web-based data capture systems, review and validation of completed forms, cross-variable consistency checks, verification calls to interviewers, supervisors, or informants, and return to field when important information required confirmation. Additional quality-control procedures were applied to anthropometric measurements, including monitoring of decimal digit preference in length/height and weight measurements and verification of extreme values for length/height, weight, and hemoglobin [43].

### 2.2. Definition of the Outcome Variable

The dependent variable used in this study was chronic undernutrition, or stunting, among children aged below 24 months. The outcome was operationalized using the official ENDI-derived variable dcronica_2, which is binary and recorded at the child level. The variable dcronica_2 equals 1 when a child is classified as chronically undernourished and 0 when the child is not classified as chronically undernourished. In the present analysis, this variable was used for children aged 0–23 months.

According to the ENDI methodological documentation [43], chronic undernutrition among children under five years is defined as insufficient length/height-for-age and is calculated using length/height-for-age standard deviations. The ENDI documentation also aligns this indicator with the WHO Child Growth Standards threshold of length/height-for-age below −2 standard deviations from the median. Therefore, dcronica_2 was treated as an official ENDI-derived binary indicator based on WHO growth standards. In this study, height-for-age z-scores were not recalculated from the raw anthropometric measurements.

The anthropometric information used by ENDI to construct chronic undernutrition indicators is collected in Form 1, Section 5 of the ENDI questionnaire, which corresponds to anthropometry [43]. The structure of the ENDI relational databases is shown in Figure 2.

For reference, Table 2 presents the standard height-for-age Z-score categories commonly used to describe chronic undernutrition severity. However, the present study did not model moderate and severe chronic undernutrition separately; it used the official binary ENDI variable dcronica_2 as provided in the database.

### 2.3. Independent Variables

The independent variables used in this study capture four analytic domains: (i) infant feeding practices, (ii) perinatal and maternal nutritional exposures, (iii) infant micronutrient supplementation, and (iv) socioeconomic and geographic context. All variables derive from the ENDI lactation module (Lactancia), the child health module (Salud en la Niñez), and household member composition data. Modules have been merged based on id_per, id_hogar, and id_mef. The relation guarantees a one-to-one mapping between children and lactation and child health modules and a many-to-one mapping between children and maternal modules. The final dataset contains all children aged 0 to 23 months (*n* = 8613) and includes 113 variables without any data duplication or deletion. The final modelling sample was subsequently defined after excluding records with missing values in the stunting outcome variable.

#### 2.3.1. Infant Feeding Practice Variables

The infant feeding practices data is sourced from the Lactancia module, where there are standardized 24-h recall measures of breastfeeding behavior, liquid consumption, complementary feeding, and food variety. Table 3 presents the key variables used in this area.

#### 2.3.2. Perinatal and Maternal Nutrition Variables

Nutritional exposures during the perinatal period are drawn from the Salud en la Niñez module and include information about pregnancy and birth history. Nutritional exposures during the perinatal period are drawn from the Salud en la Niñez module and include information about pregnancy and birth history. These variables were clinically relevant to early-life nutritional status, although completeness varied across indicators. In particular, reported birthweight information had substantial missingness after plausibility filtering and was therefore interpreted cautiously. Table 4 summarizes these variables.

#### 2.3.3. Infant Micronutrient Supplementation (“Chispaz”)

“Chispaz” is a micronutrient supplement that is provided by the Ministry of Health of Ecuador for the prevention of anemia and micronutrient deficiencies among children. It contains iron, zinc, vitamin A, C, D, and B-complex. The ENDI survey questionnaire includes a block on supplementation in which both program participation and compliance are included. The variables for Chispaz consumption are as follows:

- f2_s4i_488: Receipt of Chispaz in the past 12 months (binary).
- f2_s4i_489_a: Total number of sachets received.
- f2_s4i_489_b: Number of sachets consumed in the last seven days.

#### 2.3.4. Additional Contextual and Survey-Design Covariates

Contextual, demographic, socioeconomic, geographic, and survey-design variables were extracted from the household composition and stratification datasets and matched by id_hogar. Substantive contextual variables included child demographic characteristics, socioeconomic indicators, and geographic residence. Survey-design and data-collection variables, including expansion factors, stratification variables, and interview timing, were retained to document the structure of the ENDI data and, in some predictive specifications, were treated as non-substantive covariates. These variables were not applied as sampling weights and were not interpreted as nutritional, maternal, socioeconomic, or geographic predictors. Table 5 summarizes these variables and their analytic role.

#### 2.3.5. Operational Integration of Variables

All modules were combined based on the hierarchy used in the ENDI survey. Information pertaining to children at the level of the Lactancia and Salud en la Niñez surveys was directly concatenated based on id_per. Household characteristics were concatenated using id_hogar, while maternal information was concatenated using id_mef based on a many-to-one structure considering the possibility of having siblings. All integrity checks showed that all 8613 children aged between 0 and 23 months were maintained in the dataset with one-record frequency distributions for the child identifiers and complete preservation of maternal and household linkages.

### 2.4. Data Management and Construction of the Analytic Dataset

The analytic data file was created by combining four ENDI survey instruments, which are Personas (household roster), Lactancia (infant feeding instrument), Salud en la Niñez (children’s health instrument), and Madres en Edad Fértil (reproductive age women instrument). The complete database files were read in their semicolon-delimited format and then coded using the ENDI hierarchical identifier system.

Initially, the sample was restricted to include children who were aged between 0–23 months using the age category classification in the survey. The creation of the child-level base file included household identifiers, child identifiers, mother–child linkage variables, interview dates, survey expansion factors, survey-design variables, and contextual covariates. The cleaning process for the dependent variable (dcronica_2) involved replacement of placeholder characters with missing values.

Variables from the infant feeding dataset in the Lactancia module were selected on the basis of relevance to breastfeeding practices, liquid consumption, complementary feeding practices, and food diversity. The infant feeding dataset was then merged 1–1 based on the child identification variables (id_upm, id_viv, id_hogar, id_per) with the child dataset. This same procedure was done in the Salud en la Niñez module to merge perinatal supplementation variables, birth anthropometry, and postnatal supplementation indicators.

The variables regarding prenatal care and supplements for mothers are collected from the Madres en Edad Fértil module, using a many-to-one merge by the person identification number of the mother in the household roster. This allows each child to be matched with their respective mother’s information.

**Figure 3:**
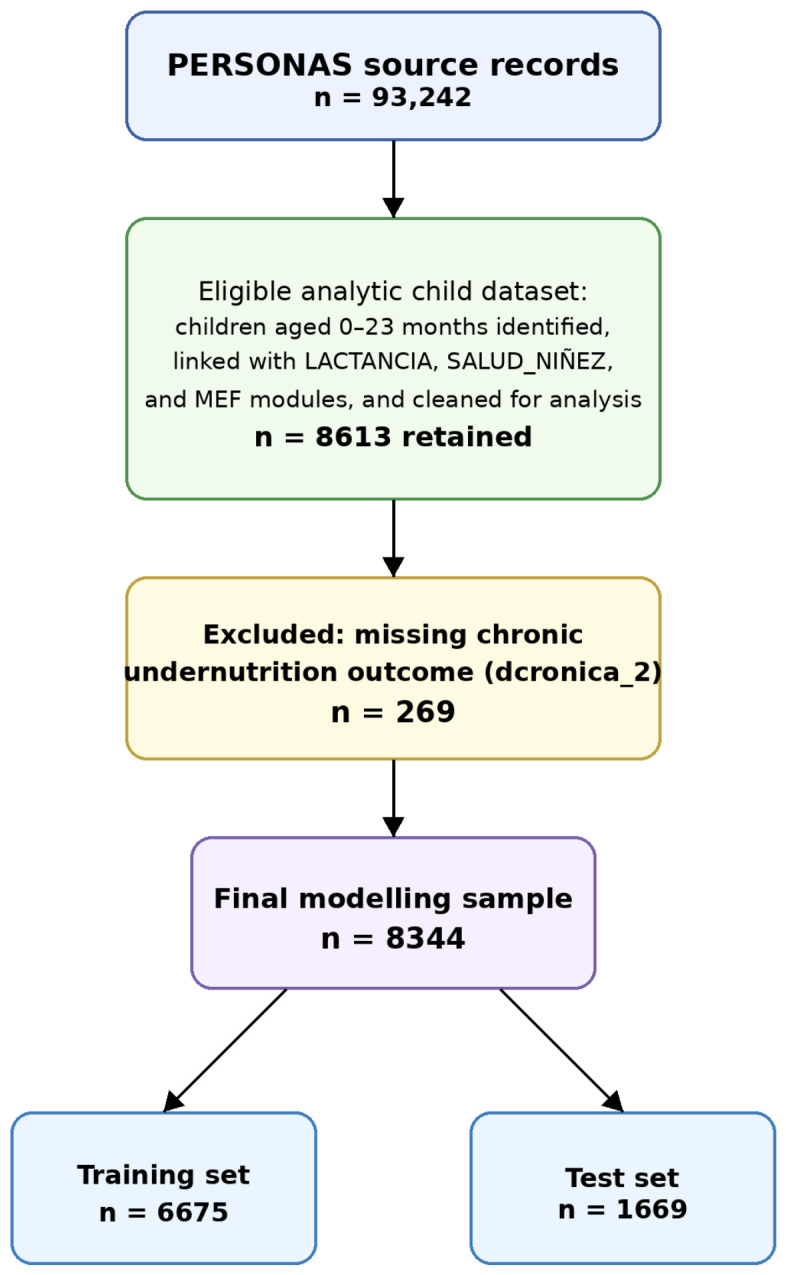
Analytic sample flow from the ENDI source records to the final modelling sample. The integration process retained all 8613 eligible children aged 0–23 months. The only record-level exclusion before modelling was due to missing chronic undernutrition status (dcronica_2), resulting in a final modelling sample of 8344 children.

Structural validity checks were done at each stage of the integration, including checking for the uniqueness of the identifier, confirming the merge cardinality (1:1 for the child modules and many:1 for the maternal modules), and evaluating the retained sample size. These checks confirmed that no observations were lost or duplicated during the merging process. The integrated child-level file retained all 8613 eligible children aged 0–23 months and included all variables necessary for the study domains of feeding, perinatal nutrition, supplementation, and socioeconomic context. Records were only excluded later if the stunting outcome variable (dcronica_2) was missing (see Figure 3).

### 2.5. Data Preprocessing and Exploratory Analysis

After the process of dataset integration, several more preprocessing steps were carried out in order to increase consistency in data and make further analysis possible. The variables which did not carry much analytical value were discarded; thus, the number of variables decreased from 113 to 111. Numerical variables, such as the target variable (dcronica_2) and birth weight (peso_nacer_g), were standardized numerically. Birth weight values outside the range of 1000–5000 g were recoded as missing for the birthweight variable only because extreme values could not be validated against gestational age or clinical records in the ENDI household survey. Children with birthweight values outside this range were not excluded from the analytic dataset or modelling sample. These preprocessing steps modified variable coding and missing-value representation but did not remove child records from the analytic file. The lower bound was selected because birthweights below 1000 g correspond to extremely low birthweight, a clinically plausible but high-risk category that would require validation against gestational age or neonatal clinical records. Such validation was not available in the ENDI household survey. The upper bound was selected because macrosomia is commonly defined using thresholds of 4000 g or 4500 g, and values above 5000 g represent extreme high birthweight values that are more likely to reflect reporting or data-entry errors in survey data. Therefore, values outside the 1000–5000 g interval were not considered biologically impossible, but were treated as extreme and insufficiently verifiable in the absence of gestational-age or clinical-record validation, and were therefore recoded as missing for birthweight analyses.

Maternal supplementation variables, including folic acid intake, iron supplementation, and Chispaz supplementation, were harmonized into standardized categorical responses (Yes/No/Unknown), and additional binary indicators were constructed for downstream analyses. Exploratory analyses included prevalence tables and graphical visualizations to examine stunting across key nutritional exposures, including maternal supplementation and low birth weight.

Distributional analyses included histograms of birth weight, prevalence bar plots, boxplots stratified by stunting status, and correlation matrices among key nutritional variables. Since birth weight had substantial missingness after plausibility filtering, peso_nacer_g was not used as a record-exclusion criterion; instead, missing birthweight values were handled within the modelling pipeline when this variable was included, and birthweight-related findings were interpreted cautiously. A missingness summary was generated for all major variables used in the analysis, including the outcome, infant feeding indicators, maternal supplementation variables, Chispaz supplementation, birthweight-related variables, and socioeconomic covariates (Table 6).

### 2.6. Feature Screening and Selection

Feature screening and selection were conducted as a hybrid process combining domain-based variable definition and data-driven screening. First, candidate predictors were defined according to the ENDI questionnaire structure and the analytic domains of interest: infant feeding practices, perinatal and maternal nutrition, infant micronutrient supplementation, and socioeconomic and geographic context. This ensured that the initial candidate set was not defined solely by statistical ranking.

Before model development, variables related to identifiers and possible target leakage were excluded from the substantive candidate feature set. Variables directly related to the construction of the anthropometric outcome, such as height-for-age z-scores or alternative stunting variables, were excluded to avoid information leakage. Survey-design and data-collection variables were handled separately, as described in the survey design considerations section, and were not interpreted as substantive nutritional, maternal, socioeconomic, geographic, or biological predictors.

Variables with very high missingness and limited analytical relevance were excluded from the general candidate predictor set. This exclusion was applied at the variable level and did not remove child records. Birthweight-related variables were treated as an exception because of their substantive relevance to early growth. Therefore, peso_nacer_g was retained when included in engineered-feature specifications, but it was not used as a record-exclusion criterion. After excluding records with missing dcronica_2, the modelling dataset contained 8344 children. Remaining missing values in predictors were handled within the modelling pipeline, using median imputation for numerical variables and most-frequent-category imputation for categorical variables when required by the corresponding model.

Mutual information was used as an exploratory screening criterion to identify variables with evidence of non-linear univariate dependence with the outcome variable, dcronica_2. This step supported dimensionality reduction in a high-dimensional survey dataset, but it was not interpreted as a measure of epidemiological importance. Because mutual information is calculated marginally, variables with weak univariate associations may still be relevant in multivariable or domain-specific contexts. For this reason, mutual information rankings were interpreted together with substantive domain relevance, missingness patterns, avoidance of outcome leakage, model performance, and SHAP-based explainability results.

The reduced feature sets were then used to compare model performance under different levels of dimensionality reduction. The Top-30 feature set was used as a reduced training dataset for baseline machine-learning comparisons, while additional reduced specifications were evaluated during CatBoost model development. Variables retained in the final predictive models should therefore be interpreted as contributing to the model’s predictive structure within the analytic sample, rather than as a definitive hierarchy of epidemiological relevance.

### 2.7. Class Distribution Assessment and Imbalance Handling

The distribution of the dependent variable (dcronica_2) was examined before model development. Among the 8613 eligible children, 269 records had missing stunting status and were excluded from modelling. The final modelling sample therefore included 8344 children, of whom 6780 were non-stunted and 1564 were stunted. This corresponded to a moderate class imbalance, with 81.3% of children in the non-stunted class and 18.7% in the stunted class.

The data were split into training and test sets using an 80:20 stratified sampling strategy to preserve the outcome distribution across both subsets. The training set included 6675 observations, with 5424 non-stunted and 1251 stunted children. The independent test set included 1669 observations, with 1356 non-stunted and 313 stunted children.

Class imbalance was addressed using model-specific weighting strategies rather than record deletion. Logistic Regression and Random Forest were trained using balanced class weights, while XGBoost used a scale-positive-weight parameter derived from the class distribution in the training set. Missing predictor values were imputed within the preprocessing pipeline after the train-test split to avoid information leakage.

### 2.8. Survey Design Considerations

ENDI is a nationally representative complex household survey and includes expansion factors and design-related variables. In the present study, expansion factors were not applied as sampling weights, and survey weights were not used in descriptive analyses, analytic-sample prevalence summaries, feature selection, model training, model evaluation, or SHAP explainability analyses. Stratification and clustering were also not explicitly modelled within the machine-learning pipeline.

Some variables related to survey design or data-collection structure, including expansion factors and interview timing, were retained in some predictive specifications as non-substantive covariates. Their inclusion should not be interpreted as incorporation of the ENDI complex survey design. These variables were not interpreted as nutritional, maternal, socioeconomic, geographic, or biological predictors. In SHAP analyses, they were grouped under a separate survey/design domain to distinguish their model contribution from that of substantive study variables.

Therefore, descriptive proportions and model-based results reported in this study should be interpreted as analytic-sample estimates and predictive patterns rather than fully survey-weighted national estimates. Class weights used during model training addressed outcome imbalance only and are distinct from ENDI sampling weights. Because the full complex survey design was not incorporated, population-level interpretation of prevalence summaries, associations, model performance, and predictor-importance rankings should be made cautiously.

### 2.9. Machine Learning Model Development

Two datasets were used in the prediction process: (i) the entire cleaned dataset and (ii) the pruned dataset including only the 30 variables identified via mutual information analysis. Models were trained on the stratified training set and evaluated on the independent test set. Predictor preprocessing, including imputation, scaling, and encoding, was fitted only on the training data and then applied to the test data.

Four machine learning algorithms were implemented for comparison: Logistic Regression, Random Forest, Extreme Gradient Boosting (XGBoost), and CatBoost. The Logistic Regression algorithm served as a linear baseline model while the remaining three machine learning algorithms belonged to the category of ensemble methods for tabular data classification.

Logistic Regression was applied via lbfgs solver up to 3000 iterations. Random Forest was applied via 500 trees with minimal leaf size of five cases. The XGBoost algorithm was applied via 500 boosting rounds with tree depth of four, learning rate of 0.03, and subsampling for observations and features. Finally, CatBoost was applied via 500 boosting rounds with tree depth of four and learning rate of 0.03.

All models were trained using imbalance-aware strategies and evaluated on the original, non-resampled test set using the default classification threshold of 0.50. Logistic Regression and Random Forest used balanced class weights, while XGBoost used the training-set class ratio to weight the minority class. Performance was measured using the accuracy, ROC-AUC, precision, F1 score, sensitivity, specificity, confusion matrix, and Brier score loss metrics.

### 2.10. Feature Engineering and Model Optimization

Additional feature engineering was conducted to derive nutritional and feeding indicators from the ENDI survey data. These included dietary diversity scores, dietary diversity adequacy indicators, breastfeeding duration, breastfeeding continuation at 12 months, low-birthweight indicators, and binary variables for maternal and child micronutrient supplementation. Socioeconomic and geographic variables were also included as contextual covariates among the candidate features. The exact source variables, coding rules, formulas, and interpretation of these engineered features are provided in Supplementary Table S2.

Several modelling datasets were evaluated during model development: (i) engineered features only, (ii) the mutual-information based Top-30 feature dataset, referred to as TOP30_MI, (iii) combined engineered and mutual-information based features, and (iv) the full cleaned dataset, referred to as FULL_CLEAN. Logistic Regression, Random Forest, XGBoost, and CatBoost were first evaluated as baseline models using the Top-30 feature dataset and the full cleaned dataset. Logistic Regression, Random Forest, and XGBoost were implemented using preprocessing pipelines that included imputation, standardization of numerical variables, and one-hot encoding of categorical variables. CatBoost was trained using its native support for categorical predictors.

Baseline models were defined as non-Optuna models trained using fixed, prespecified hyperparameter settings. These models were used for initial comparison across the Top-30 feature dataset and the full cleaned dataset. Baseline classification metrics were calculated on the held-out test set using the default probability threshold of 0.50. The preprocessing steps, class-imbalance handling, and fixed model specifications used for these baseline comparisons are reported in Supplementary Table S3.

Following the baseline comparisons, additional optimized specifications were evaluated using threshold tuning, class-weighting strategies, feature-engineered variables, and reduced feature sets. These experiments included the TOP30_MI Logistic Regression model, the FULL_CLEAN XGBoost model, and the FULL_CLEAN CatBoost Native model. Model performance was evaluated on the same held-out test set using accuracy, AUC, sensitivity, specificity, precision, F1-score, and Brier score where applicable.

Hyperparameter optimization was then conducted using Optuna for the final optimized specifications reported as *Optimized TOP30 Logistic Regression* and *Optimized FULL CLEAN CatBoost Native*. Optuna used a Tree-structured Parzen Estimator sampler with a fixed random seed of 42. Optimization was performed only within the training data after the stratified train-test split; the independent test set was not used during tuning and was reserved for final model evaluation.

Within the training set, five-fold stratified cross-validation was used. The optimization direction was set to maximize the mean cross-validated objective score. For each trial and validation fold, predicted probabilities were generated and an internal threshold search was conducted over thresholds from 0.05 to 0.70 in increments of 0.02. The fold-level objective combined the minority-class $F_{1}$ score and balanced accuracy (BA) as follows:

$$\mathrm{Objective}\;\mathrm{score}=0.70\times F_{1}+0.30\times \mathrm{BA}.$$

The final objective value for each Optuna trial was calculated as the mean of this score across the five validation folds. This objective was selected because the outcome was moderately imbalanced and overall accuracy alone would not adequately reflect performance for the chronically undernourished class.

The number of Optuna trials was prespecified. Logistic Regression was optimized using 30 trials, while CatBoost Native was optimized using 60 trials. Logistic Regression stopped after the prespecified number of trials. CatBoost Native additionally used early stopping during cross-validation, with training stopped after 100 rounds without improvement on the validation set. After hyperparameter optimization, the selected model configurations were retrained on the training data and evaluated once on the held-out test set. The hyperparameter search spaces used for the Optuna procedures are reported in Supplementary Table S4.

Additional ensemble-learning experiments were conducted using probability blending of the optimized Logistic Regression and CatBoost Native models. Two weighted blending configurations were retained for reporting: 70% Logistic Regression/30% CatBoost, which prioritized overall accuracy and specificity, and 85% Logistic Regression/15% CatBoost, which showed the most balanced ensemble performance. These ensemble specifications were evaluated using the same held-out test set and classification metrics used for the individual models.

For the final ensemble-learning comparison, approximate 95% confidence intervals for AUC estimates were calculated using a large-sample normal approximation based on the held-out test-set AUC and the number of stunted and non-stunted children in the test set. The test set included 313 stunted children and 1356 non-stunted children. These intervals were added to quantify uncertainty around model discrimination for the final ensemble-learning models, which represented the best-performing model configurations retained for comparison.

To improve interpretability of the final predictive model, SHAP analysis was performed on the optimized CatBoost model. SHAP summary plots and global feature-importance rankings were used to examine the contribution of nutritional, maternal, socioeconomic, geographic, and survey/design variables to the model’s predictive structure. Variables related to survey design or data-collection context were interpreted as non-substantive covariates and were not treated as sampling weights or as nutritional, maternal, socioeconomic, geographic, or biological predictors. SHAP results were interpreted as model-based predictive patterns within the analytic sample, rather than as causal or epidemiological determinants of chronic undernutrition. SHAP feature importance was summarized using mean absolute SHAP values. Predictors were ranked according to their average absolute SHAP contribution across the observations used for SHAP analysis. The ranked list of the highest-contributing predictors, including exact mean absolute SHAP values, is reported in Supplementary Table S5.

## 3. Results

A total of 8613 children aged 0–23 months were identified in the ENDI PERSONAS module and included in the initial child-level dataset. Linkage with the LACTANCIA, SALUD_NINEZ, and maternal MEF modules did not result in any loss of observations, and all 8613 children were retained after the integration process. After converting placeholder values in the outcome variable to missing values, 269 children were excluded because stunting status (dcronica_2) was unavailable. Therefore, the final analytic and modelling sample included 8344 children, corresponding to 96.9% of the eligible sample. For model development, the data were divided using an 80:20 stratified train-test split, resulting in 6675 observations for training and 1669 observations for testing.

In the analytic ENDI sample, stunting was observed in approximately one in every five children. The following sections present descriptive statistics for perinatal, maternal, and infant nutrition-related variables, followed by exploratory analyses used to inform the machine-learning modelling process. These initial findings describe birth anthropometry and supplementation patterns.

**Figure 4:**
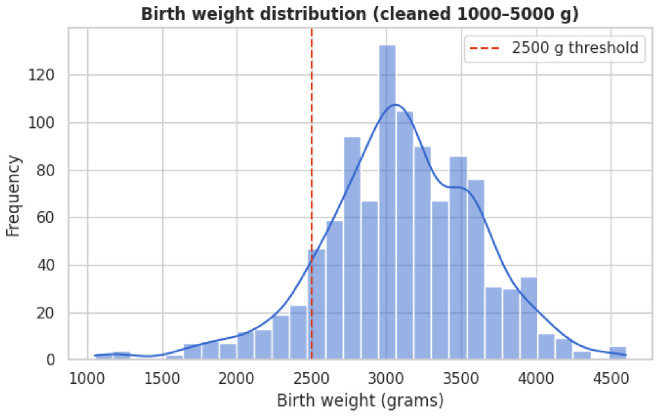
Distribution of birth weight among children aged 0–23 months after recoding values outside 1000–5000 g as missing. The vertical dashed line marks the low-birth-weight threshold of 2500 g. The blue curve represents a kernel density estimate, which provides a smoothed visual summary of the birth-weight distribution.

The distribution of birth weight for children aged between 0–23 months is depicted in Figure 4. After recoding out-of-range birthweight values as missing for the birthweight variable only, the distribution was approximately normal, with a mean value of 3094 g (SD = 528 g). A smaller proportion of children had birth weight values below 2500 g, which is defined as low birth weight (LBW) and is indicated by the dashed line. The distribution shows variability in birth anthropometry within the analytic ENDI sample. Because low birth weight has been associated with subsequent linear growth faltering, birthweight-related variables may capture early-life vulnerability associated with stunting.

**Figure 5:**
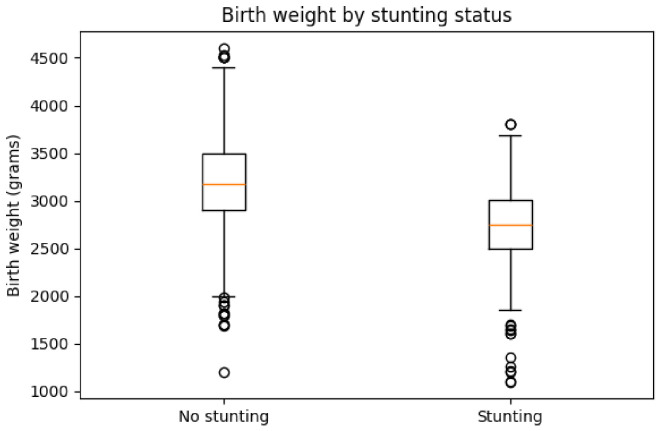
Birth weight distribution by stunting status among children aged 0–23 months. Children with stunting showed a lower birthweight distribution and a greater concentration of values near the low-birth-weight threshold.

Figure 5 illustrates the distribution of birth weight among children with and without stunting. The results indicate a descriptive difference in birth weight between groups, with non-stunted children showing a median birth weight around 2800–2900 g, whereas children classified as stunted showed a lower median birth weight. A greater proportion of values among stunted children were closer to or below 2500 g, which represents the low birth weight threshold. The interquartile range was also lower among stunted children, indicating that lower birthweight values were more common in this group. These findings are consistent with an inverse descriptive association between birth weight and stunting, although birthweight-related results should be interpreted cautiously given the missingness in this variable.

Among children with valid birthweight and non-missing stunting outcome data, mean birthweight was lower among children classified as stunted than among non-stunted children (Table 7). Non-stunted children had a mean birthweight of 3174.0 g (95% CI: 3141.2–3206.9), whereas stunted children had a mean birthweight of 2702.4 g (95% CI: 2617.8–2786.9).

In the unadjusted comparison, children classified as stunted had a mean birthweight 471.7 g lower than non-stunted children (95% CI: −562.0 to −381.3; p<0.001). After adjustment for child age group, area, region, wealth quintile, poverty status, unmet basic needs, maternal folic-acid supplementation, and maternal iron supplementation, the adjusted mean difference was −465.9 g (95% CI: −558.8 to −373.1; p<0.001). These comparisons were based only on children with valid birthweight information and should be interpreted cautiously because birthweight had substantial missingness (Table 8).

**Figure 6:**
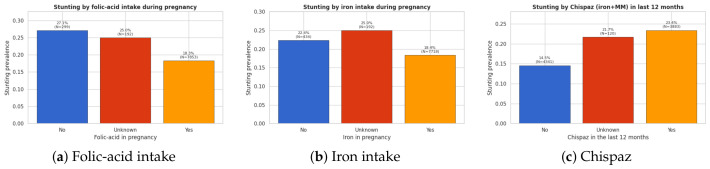
Analytic-sample stunting prevalence by maternal and child micronutrient supplementation.

Within the analytic sample, stunting prevalence varied across maternal and child micronutrient supplementation categories (Figure 6). For maternal folic acid supplementation, children whose mothers reported no use of folic acid had a stunting prevalence of 27.1%, whereas children with missing maternal supplementation information had a prevalence of 25.0%, and children whose mothers reported folic acid use had a prevalence of 18.3% (Figure 6a). A similar descriptive pattern was observed for maternal iron supplementation (Figure 6b), with prevalence values of 22.4% among children whose mothers reported no iron supplementation, 25.0% among those with missing information, and 18.3% among those whose mothers reported iron supplementation. By contrast, child supplementation with Chispaz (iron + multiple micronutrients) showed a different pattern (Figure 6c), with lower stunting prevalence among children who did not use the supplement (14.5%) and higher prevalence among children who were using it (23.4%). This finding is more plausibly interpreted in light of the programmatic use of Chispaz among nutritionally vulnerable children than as evidence of an adverse role of the supplement itself.

**Figure 7:**
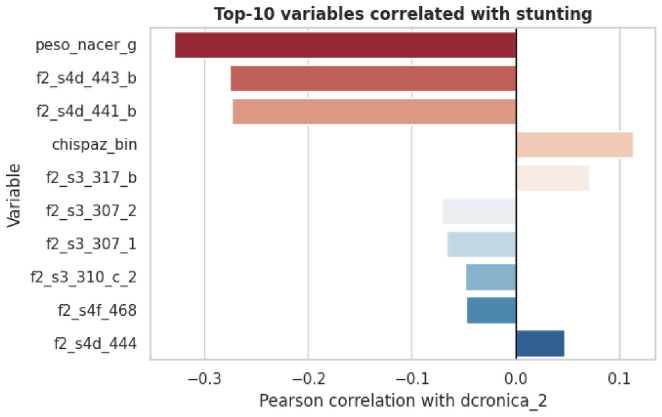
Top-10 variables most strongly correlated with stunting (dcronica_2). Negative values indicate inverse correlations, while positive values indicate positive correlations. Bar colors represent the relative strength of the correlations, with blue tones indicating weaker correlations and reddish tones indicating stronger correlations.

Variables related to birth anthropometry showed the strongest correlations with stunting within the analytic sample (Figure 7). Birth weight had the highest inverse correlation with stunting (r=−0.33), while the two variables related to birth length had similar inverse correlations (r≈−0.27). These correlations indicate modest descriptive associations and should not be interpreted as evidence of causal importance. The pattern is consistent with previous evidence linking restricted fetal growth with later linear growth faltering in early childhood.

Among nutritional variables, Chispaz supplementation (chispaz_bin) showed a small positive correlation with stunting (r=0.11). This pattern should be interpreted cautiously because supplementation programs may be more common among children already identified as nutritionally vulnerable or more connected to health services. Indicators of feeding practices showed even weaker correlations (|r|≈0.05–0.07), indicating limited linear association between individual IYCF behaviors and stunting in the analytic sample.

In general, the results presented above suggest the value of incorporating non-linear and interaction models in the next steps of the study, since bivariate correlation can account for only a small part of multi-dimensional interactions.

The target variable (dcronica_2) showed moderate class imbalance in the final modelling sample. Among the 8344 children with non-missing stunting outcome data, 6780 were non-stunted and 1564 were stunted, corresponding to 81.3% and 18.7% of the modelling sample, respectively. This distribution was preserved in the stratified train-test split: the training set contained 5424 non-stunted and 1251 stunted children, while the test set contained 1356 non-stunted and 313 stunted children.

Baseline machine learning algorithms showed modest to moderate performance for stunting classification (Table 9). In the reduced Top-30 features dataset, the highest accuracy was obtained with Random Forest at 79.9%, followed by XGBoost and CatBoost with 77.3% each. The lowest accuracy was obtained with Logistic Regression at 74.2%, but its sensitivity was considerably higher at 37.1%, suggesting greater detection of stunted cases. In the entire cleaned dataset, accuracy improved slightly with Logistic Regression and Random Forest up to 80.4%. However, this slight increase in accuracy was accompanied by extremely low sensitivity for the minority stunted class, especially for Logistic Regression, where no positive stunting cases were detected in the independent test data.

Further experiments involving feature engineering, threshold tuning, and class-weighting yielded improvements in predictive performance compared to the baseline models (see Table 10). The strongest results among the optimized models were obtained using Logistic Regression on the mutual-information based Top-30 features (TOP30_MI). This model achieved the highest accuracy (81.4%), precision (48.1%), and F1-score (0.419) across all optimized models.

Among the optimized models, XGBoost with the full cleaned dataset showed the most balanced combination of accuracy (73.7%), sensitivity (42.9%), and specificity (80.5%), indicating improved detection of the minority stunted class while maintaining moderate accuracy and specificity. CatBoost with the full cleaned dataset had the highest sensitivity (71.4%), indicating greater detection of stunted children after threshold optimization.

In conclusion, feature selection based on mutual information combined with threshold optimization improved classification performance, while full-dataset ensemble models showed higher sensitivity for the minority stunted class.

Optimized hyperparameters using Optuna resulted in varied changes in predictive performance (Table 11). The Logistic Regression model optimized with the Top-30 features showed the strongest results among these models, with accuracy of 70.1%, sensitivity of 54.3%, and F1-score of 0.396. In comparison with other baseline models, this model showed improved detection of the minority stunted class while maintaining moderate discrimination.

The optimized CatBoost model trained on the whole cleaned dataset resulted in higher overall accuracy (74.2%) and a lower Brier score (0.171), suggesting better probability calibration relative to the other optimized models. Nevertheless, the optimized CatBoost model still showed lower sensitivity (37.1%) compared to the optimized Logistic Regression model, indicating persistent difficulty in detecting the minority stunted class.

In general, the Optuna optimization step suggested that hyperparameter tuning and feature selection may improve the trade-off between predictive accuracy and minority class detection, especially for the Logistic Regression model trained on mutual information features.

Additional classification experiments were conducted using an ensemble learning approach, where two optimized models, Logistic Regression and CatBoost, were used together (Table 12). The highest prediction accuracy among the ensemble models was obtained with a weighted blending model using 70% of Logistic Regression and 30% of CatBoost probabilities. This ensemble model attained an accuracy of 83.0%, as well as high specificity (94.9%) and the highest precision among the evaluated models (55.6%). Its AUC was 0.622, with an approximate 95% confidence interval of 0.586–0.658.

However, the weighted blend configuration of 85% Logistic Regression and 15% CatBoost showed the most balanced discrimination performance among the ensemble models, with the highest balanced accuracy (64.7%), the highest F1-score (0.416), and a more even balance between sensitivity and specificity. Its AUC was 0.624, with an approximate 95% confidence interval of 0.588–0.660. The optimized Logistic Regression model on its own yielded the highest sensitivity (54.3%), indicating greater detection of the minority stunted class than the ensemble models.

Overall, the ensembles performed competitively with the individual models, balancing overall accuracy and minority-class detection across the evaluated configurations. Nevertheless, the overlapping AUC confidence intervals support a cautious interpretation of model discrimination, which remained modest across the final evaluated models.

**Figure 8:**
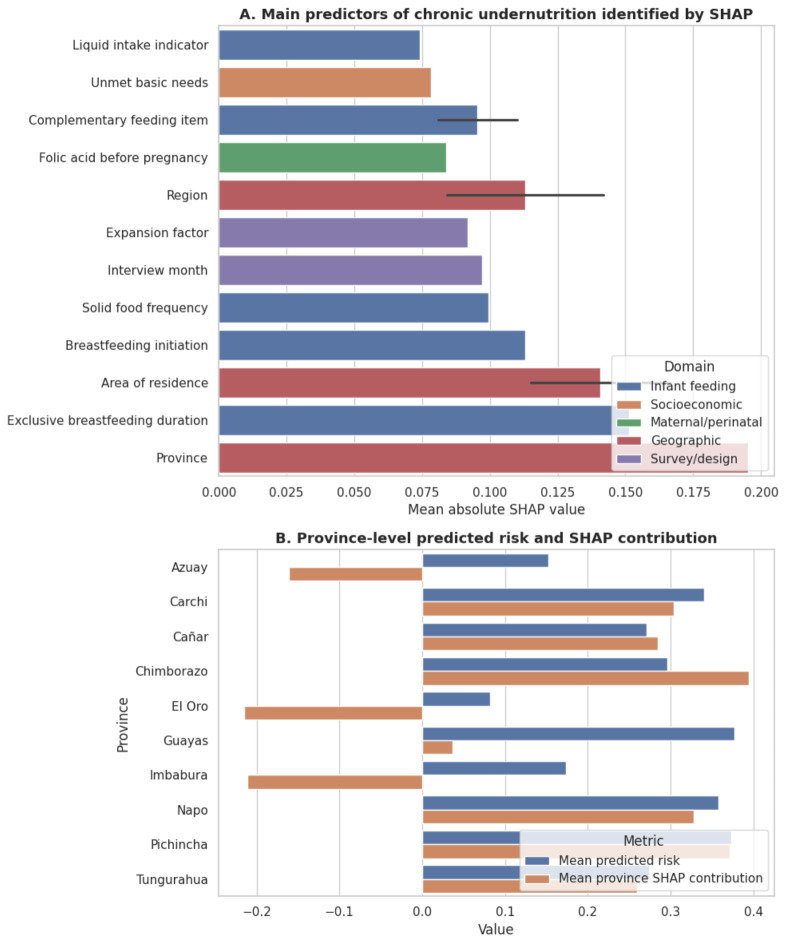
Combined SHAP explainability analysis for the optimized CatBoost model. Panel (**A**) presents the main features and covariates contributing to stunting prediction, grouped by domain according to their mean absolute SHAP contribution across the test set. Variables classified under the survey/design domain, such as expansion factors and interview timing, were included as non-substantive model covariates and were not applied as sampling weights. Panel (**B**) compares average predicted stunting probabilities with average province-level SHAP contributions, illustrating geographic variation within the predictive structure identified by the model.

SHAP analysis was applied to the optimized CatBoost model to assess the contribution of nutritional, maternal, socioeconomic, and geographic features, as well as survey/design-related non-substantive covariates, to stunting prediction (Figure 8). The SHAP values matrix included 194 observations and 119 predictor variables.

The main contributors to model prediction included province and area of residence (prov_x, area_x), breastfeeding and complementary feeding indicators, feeding frequency, unmet basic needs (nbi_1), maternal folic acid supplementation before pregnancy (f2_s4b_408), and survey/design-related non-substantive covariates, including expansion factors and interview timing. These latter variables were interpreted as reflecting survey structure or data-collection context, rather than as nutritional, maternal, socioeconomic, geographic, or biological predictors of stunting.

Furthermore, geographical variation was observed across provinces. The three provinces with the highest average predicted probabilities from the optimized CatBoost model were Guayas, Pichincha, and Napo, while Chimborazo had the greatest positive average SHAP contribution at the province level. Other provinces, such as El Oro, Imbabura, and Azuay, had negative average SHAP values. Nutritional and feeding variables also remained among the substantive contributors to model prediction.

The exact mean absolute SHAP values for the highest-ranked predictors are provided in Supplementary Table S5. Province and area of residence had the largest average SHAP contributions, followed by feeding-related variables, regional indicators, unmet basic needs, and survey/design-related covariates. Variables related to survey design or data-collection structure, such as expansion factors and interview timing, were retained in the ranked SHAP output for transparency but were not interpreted as substantive nutritional, maternal, socioeconomic, geographic, or biological predictors.

## 4. Discussion

Stunting remained common among Ecuadorian children aged 0–23 months in the analytic sample, affecting 18.7% of children included in the final modelling sample. The analytic workflow retained most of the eligible ENDI sample, with 8344 of 8613 children included after excluding only records with missing stunting status. This indicates that record exclusion was limited at the outcome level. However, predictor-level missingness, particularly for birthweight-related variables, still requires cautious interpretation. The repeated prominence of birth variables suggests that early-life anthropometric vulnerability was represented in the predictive structure of the data.

In this respect, the analysis of birthweight distribution provides information about prenatal and early-life anthropometric conditions within the analytic sample. While the cleaned distribution showed a mean birth weight of 3094 g, the presence of observations near the 2500 g low-birth-weight threshold indicates variability in birth anthropometry. Considering the established association between low birth weight and poorer linear growth, this finding suggests that some stunting patterns observed in the data may be related to prenatal or perinatal vulnerability, in addition to postnatal feeding and nutrition-related conditions. Thus, feeding practices and supplementation indicators should be interpreted within a broader early-life context that includes birth anthropometry and prenata vulnerability.

This interpretation is further supported by the birthweight distribution among children with stunting, who showed lower median birth weights and a greater concentration of observations near the low-birth-weight threshold. In the exploratory analysis, birth weight showed the strongest inverse correlation with stunting (r=−0.33), while similar inverse correlations were observed for birth length measures (*r* ≈ −0.27). These correlations suggest that early-life anthropometric variables were descriptively associated with stunting in the analytic sample, although they should not be interpreted as evidence of causal importance.

The analysis of supplementation patterns suggests that maternal nutrition-related practices during pregnancy were associated with differences in stunting prevalence within the analytic sample. Stunting prevalence was lower among children whose mothers reported folic acid supplementation during pregnancy (18.3%) than among those whose mothers did not report supplementation (27.1%). A similar pattern was observed for maternal iron supplementation, with lower stunting prevalence among children whose mothers reported iron supplementation (18.4%) than among those whose mothers did not (22.4%). These findings suggest that maternal micronutrient supplementation variables were associated with stunting in the first two years of life, although they should not be interpreted as evidence of a protective causal effect.

By contrast, the higher prevalence of stunting among children taking Chispaz supplements (23.4%) compared with non-supplemented children (14.5%) is more consistent with program targeting or confounding by indication than with an adverse nutritional association of the supplement itself. Children receiving Chispaz may have been more likely to have been identified as nutritionally vulnerable or to have had greater contact with health services. This distinction is important when interpreting supplementation variables in predictive models, where associations may reflect program eligibility, care-seeking patterns, healthcare access, or prior vulnerability rather than independent nutritional effects.

Despite low correlations between feeding practice indicators and stunting ((|*r*| ≈ 0.05)–(0.07)), these findings are more likely to reflect the multidimensional nature of stunting than a lack of relevance of feeding-related indicators. The results suggest that feeding practices, when considered individually through bivariate correlations, showed limited linear associations with stunting, while their predictive contribution may depend on broader prenatal, socioeconomic, environmental, and behavioral conditions.

This interpretation is consistent with the broader literature on infant and young child feeding, which emphasizes that feeding indicators capture standardized practices but not the full context in which those practices occur. WHO/UNICEF infant and young child feeding indicators provide standardized measures of breastfeeding, dietary diversity, meal frequency, and complementary feeding, but these indicators do not fully capture maternal attitudes, perceived breastfeeding support, cultural norms, household constraints, healthcare counselling, or decision-making processes around feeding [16]. Recent work on breastfeeding-related attitudes and functionality appreciation further suggests that positive perceptions of body functionality may be associated with breastfeeding intentions, highlighting the relevance of psychosocial factors in understanding breastfeeding behavior [44]. Evidence on complementary feeding also indicates that feeding practices are shaped by caregiver knowledge, food availability, affordability, cultural acceptability, family support, and perceived child needs [45,46]. Therefore, the weak bivariate correlations observed for individual feeding indicators in the present study should not be interpreted as evidence that feeding practices are unimportant, but rather as an indication that these practices are embedded within broader maternal, household, healthcare, and social contexts.

When taken together, these results support the application of nonlinear and interaction-focused models in further analysis. The low explanatory power of bivariate relationships suggests that stunting is better understood as part of a multidimensional pattern involving prenatal, nutritional, socioeconomic, behavioral, and contextual factors rather than as the result of isolated individual predictors.

The class imbalance found in the data was moderate and was reflected in the performance of baseline models, especially concerning their sensitivity towards the minority class. Although many models showed relatively high overall accuracy rates, this was often at the cost of lower sensitivity in identifying stunted children. For instance, Logistic Regression fitted using the whole cleaned data had an accuracy rate of 80.4%, but it could not identify any positive stunting cases (sensitivity = 0.000) in the test set. Similarly, Random Forest showed almost the same accuracy rate (80.4%) but with low sensitivity (0.086).

Logistic Regression on Top-30 features had higher sensitivity (37.1%) and the highest AUC among the baseline classifiers (0.656). However, its overall accuracy (74.2%) was slightly lower compared to those of the other classifiers. Similarly, CatBoost on the whole cleaned dataset had the lowest Brier score (0.149) and the highest AUC (0.661) among the evaluated ensemble classifiers, although this AUC still indicates modest discrimination in the presence of class imbalance.

As a whole, the baseline models indicated that different algorithms captured different aspects of stunting prediction. Although some ensemble approaches showed higher discrimination, overall discrimination remained modest, and more sensitive models were better able to detect the minority stunted class. These results suggest that stunting was represented in the predictive structure through overlapping biological, nutritional, and socioeconomic factors.

Following optimization, the models showed an improved balance between overall discrimination and minority class detection. The Logistic Regression model based on the TOP30_MI dataset showed the strongest performance among the optimized models, with 81.4% accuracy, as well as the highest precision (48.1%) and F1-score (0.419). It is worth mentioning that this improvement was obtained by using a smaller subset of features, which indicates that the selected features may have provided more informative predictive signal for stunting.

On the other hand, the ensemble methods showed greater sensitivity for identifying stunted children, highlighting the trade-off between minority class detection and overall predictive performance in stunting classification. Models that improved sensitivity often did so at the expense of specificity, reflecting the difficulty of accurately detecting relatively infrequent outcomes in imbalanced datasets.

The improvements observed after threshold optimization, feature engineering, and hyperparameter tuning suggest that nonlinear relationships, interaction-aware modelling, and calibration procedures may contribute to stunting prediction. Nevertheless, the strong relative performance of the optimized Logistic Regression model indicates that part of the predictive information can still be captured through relatively stable linear relationships among nutritional, maternal, and socioeconomic variables.

The Optuna optimization stage further highlighted the need to balance overall predictive accuracy with minority class detection. While optimization improved calibration and discrimination for some models, differences in sensitivity across approaches suggested persistent challenges in reducing the tendency toward the majority class. Overall, these findings support the value of combining feature selection, threshold tuning, and optimization strategies to improve model performance in stunting prediction.

The increase in minority class detection following feature selection and hyperparameter tuning suggests that stunting prediction improved when biological, nutritional, and socioeconomic predictors were considered together. However, this improvement should be interpreted as enhanced model performance within the analytic sample rather than as evidence of readiness for individual-level risk classification.

The persistent competitive performance of Logistic Regression suggests that relatively simple models can capture part of the predictive structure, even when compared with more advanced ensemble-learning approaches. However, the results also indicate that more flexible modelling strategies may help summarize multidimensional predictive patterns related to stunting, although their modest discrimination limits their use for individual-level identification of nutritionally vulnerable children.

The ensemble-learning experiments showed that combining complementary model architectures provided different trade-offs between sensitivity and specificity. The 70% Logistic Regression/30% CatBoost model provided the highest overall accuracy (83.0%) and specificity (94.9%), but sensitivity remained low (28.6%). By contrast, the 85% Logistic Regression/15% CatBoost model showed the most balanced performance among the ensemble configurations, achieving the highest balanced accuracy (64.7%) and F1-score (0.416), along with higher sensitivity (45.7%). Therefore, models with a better balance between discrimination metrics may be more informative than models that prioritize high overall accuracy alone.

The SHAP explainability analysis provided further insight into the multidimensional predictive structure of stunting. Variables related to exclusive breastfeeding, breastfeeding initiation time, complementary feeding practices, and feeding frequency appeared consistently among the features contributing to prediction in the CatBoost model. This finding suggests that recommended infant feeding practices were represented in the model’s predictive structure, although these SHAP contributions should not be interpreted as evidence that such practices causally reduce stunting risk.

Maternal folic acid supplementation also showed relevant SHAP contributions, consistent with the descriptive patterns observed in the earlier analyses. However, the contribution of geographical location, area of residence, and unmet basic needs indicates that nutritional practices alone do not account for stunting prediction. Instead, the explainability analysis suggests that feeding practices, maternal nutrition, and socioeconomic inequalities were jointly represented within the predictive framework.

On the whole, the SHAP analyses support the use of explainable ensemble-learning models to summarize heterogeneous and potentially nonlinear predictive associations that may not be fully captured by traditional linear analyses. Instead of pointing to a single dominant predictor, the model suggests that stunting prediction was distributed across prenatal, nutritional, socioeconomic, and structural factors represented within the analytic sample.

To place these findings in context, the results based on the ENDI analytic sample should be considered in relation to the broader empirical literature on childhood stunting. The present study identified predictive patterns involving birth anthropometry, maternal supplementation, infant feeding indicators, socioeconomic conditions, and geographic context. These domains have also been examined in prior studies across diverse epidemiological and methodological settings. Comparing the current findings with previous research helps clarify where the observed patterns are consistent with existing evidence, where differences may arise from context or methodology, and how explainable machine-learning models add value by summarizing multidimensional predictive structures.

The results obtained in the current study are consistent with, yet further enrich, the literature regarding the correlates of stunting during infancy, as summarized in Table 1. Prior research has indicated socioeconomic disadvantage, poor infant nutrition, and low birth weight as significant correlates of poor child growth [33,38,39]. Many of these studies have relied on conventional multivariable regression models to estimate adjusted associations between individual risk factors and anthropometric outcomes. The present findings are broadly consistent with those epidemiologic approaches in identifying birth anthropometry, socioeconomic disadvantage, maternal supplementation, and infant feeding indicators as relevant domains. However, rather than estimating adjusted odds ratios for individual exposures, the present study used explainable machine-learning models to examine how these domains jointly contributed to stunting prediction within a multidimensional predictive framework. This distinction is important because the objective of the analysis was predictive and exploratory rather than inferential or causal.

In comparison to past machine learning studies [34,35], the current study contributes methodologically through the use of ENDI data, the incorporation of prenatal and postnatal nutrition-related variables, and SHAP-based explainability analysis. Compared with prior regression-based studies in Ecuador [36,42], geographic location, socioeconomic status, breastfeeding practices, complementary food intake, maternal supplementation, and birth-related factors were among the substantive features contributing to prediction in the SHAP explainability analysis. Province-level analyses showed geographic variation, with Chimborazo showing the strongest positive SHAP contribution among provinces, while Guayas, Pichincha, and Napo exhibited the highest average predicted probabilities generated by the optimized CatBoost model. These findings suggest that stunting was represented within the machine-learning framework as a multidimensional phenomenon involving both contextual and nutritional factors during early life. Nevertheless, the models consistently demonstrated stronger predictive performance for the majority non-stunted class than for the minority stunted class, despite the implementation of imbalance-aware modelling strategies and threshold optimization procedures.

The lower stunting prevalence observed among children whose mothers reported iron and folic acid supplementation is consistent with previous studies indicating that maternal micronutrient supplementation is associated with lower prevalence of low birth weight and other adverse pregnancy outcomes [40]. However, as noted above, supplementation variables require cautious interpretation in cross-sectional data because they may also reflect antenatal care access, health-service contact, maternal nutritional status, and program eligibility. Similarly, the higher prevalence of stunting among children receiving Chispaz supplementation is more likely to reflect the targeting of nutritionally vulnerable children by the intervention rather than an adverse association of the supplement itself.

Overall, this study adds to the current literature by examining prenatal, perinatal, and postnatal nutrition-related factors within the ENDI analytic sample using explainable machine-learning techniques. The findings suggest that stunting in the 0–23-month age range was represented by multidimensional predictive patterns involving birth anthropometry, maternal supplementation, infant feeding indicators, socioeconomic conditions, and geographic context. In this sense, the ENDI-based machine-learning approach provides a methodological complement to previous epidemiologic research on early-life child growth and stunting.

### Limitations and Future Work

There are several limitations that must be taken into account in the interpretation of these results. Firstly, the cross-sectional nature of the ENDI survey precludes causal inference and prevents the determination of temporal ordering, especially concerning supplementation measures. This is exemplified by the positive association between the use of Chispaz and stunting, where reverse causation, program targeting, healthcare access, and confounding by indication remain valid concerns. Secondly, although the revised analytic workflow retained most eligible children and the final modelling sample included 8344 of 8613 children, predictor-level missingness remained relevant, particularly for birthweight-related variables. This missingness may have affected the interpretation of birth anthropometry and may still introduce selection-related bias that cannot be fully ruled out.

Thirdly, ENDI is a nationally representative complex household survey, but expansion factors were not applied as sampling weights, and clustering and stratification were not explicitly modelled within the machine-learning pipeline. Therefore, the descriptive proportions, associations, model performance, and SHAP-based rankings should be interpreted as analytic-sample findings and predictive patterns rather than fully survey-weighted national estimates. Some survey/design-related variables were retained in some predictive specifications as non-substantive covariates; however, their inclusion should not be interpreted as incorporation of the ENDI complex survey design. In addition, SHAP values were interpreted as feature contributions to model prediction rather than as evidence of causal effects, biological mechanisms, or substantive importance.

Fourthly, some variables were based on caregiver-reported information, including feeding practices and supplementation indicators. These measures may be affected by recall bias, reporting error, and day-to-day variation in infant feeding practices. In addition, several clinical variables, such as morbidity events, maternal anthropometry, and biochemical measurements, were missing or incomplete, limiting further investigation of biological processes beyond feeding and supplementation practices. Residual confounding due to unmeasured or imperfectly measured socioeconomic, clinical, and healthcare-access factors also cannot be excluded.

Lastly, although machine-learning methods were useful for summarizing multidimensional predictive patterns, overall discrimination remained modest, with AUC values around 0.62–0.66 across the main model specifications. These values indicate limited predictive utility for individual-level classification, and statistical or predictive signal should not be interpreted as readiness for deployment. The models were evaluated using internal train-test splits but were not externally validated in an independent dataset, survey wave, or population. Future work should incorporate the full complex survey design, evaluate survey-weighted and design-aware modelling strategies, assess external validation, and examine whether longitudinal data can better distinguish temporal ordering between feeding, supplementation, healthcare access, and stunting.

Future research could address these limitations by using longitudinal datasets able to track nutritional, clinical, and household conditions across the first 1000 days of life. Incorporation of clinical, biomarker, geospatial, and environmental data could also provide a more complete understanding of the biological and structural conditions associated with stunting. Methodologically, future studies could evaluate causal machine-learning approaches, survey-weighted predictive models, and externally validated ensemble methods.

## 5. Conclusions

The current research offers an analysis of nutritional, maternal, and socioeconomic factors related to chronic undernutrition among Ecuadorian children aged 0–23 months using explainable machine learning techniques on the ENDI analytic sample. In general, the results suggest that stunting is represented through combined predictive patterns involving prenatal, nutritional, and structural vulnerabilities.

Infant and young child feeding behaviors were associated with stunting, although their individual linear relationships with the outcome were weak when assessed separately. However, indicators related to breastfeeding behavior, complementary feeding, and feeding frequency contributed to prediction in the SHAP explainability analysis, suggesting that feeding-related variables were represented within the broader multidimensional predictive structure.

Maternal iron and folic acid supplementation were consistently associated with lower stunting prevalence and lower predicted probability of stunting, emphasizing the relevance of prenatal nutritional conditions during early growth and development. On the other hand, the positive correlation found for Chispaz supplementation was more consistent with the targeting of nutritionally at-risk children rather than with an adverse nutritional association of the supplement itself.

Among the features contributing to stunting prediction, birthweight-related variables showed strong associations with stunting, although these findings should be interpreted cautiously due to the high level of missingness in birthweight data. Socioeconomic factors, maternal supplementation, and feeding variables also contributed to the predictive structure. SHAP analysis further showed that machine learning methods can help summarize the multidimensional predictive patterns associated with stunting vulnerability.

When considered together, these findings add to the existing body of knowledge on early-life undernutrition in Ecuador by combining prenatal, perinatal, and postnatal factors within a common analytical framework specific to the critical 0–23-month window. The results suggest that future nutrition strategies should not be confined to infant feeding practices alone, but should also consider maternal nutrition and socioeconomic inequality.

## Figures and Tables

**Table 1:** State of the art on child growth, nutrition, and stunting.

Article	Country/Data Source	Population	Approach	Main Gap Addressed by the Present Study
[33]	Ethiopia; public health centers	395 mother–child pairs; children <24 months	Bayesian logistic regression; latent class analysis	No ML/XAI approach; limited standardized IYCF and biomarker indicators.
[34]	China; child health clinic data	9581 children aged 0–36 months	LASSO; logistic regression; nomogram validation	Predictive modelling used, but limited IYCF detail and no Latin American survey context.
[35]	UNICEF–WHO–WB data; simulated dataset	Children aged 0–5 years; synthetic data	XGBoost; clustering; PCA; reinforcement learning	Based on simulated data; no WHO anthropometric z-scores or IYCF indicators.
[36]	Ecuador; rural Cotopaxi cohort	125 children; mean age 34 months	Longitudinal regression; mediation analysis	Ecuador-specific, but small non-national sample and no ML/XAI modelling.
[37]	Ecuador; ENSANUT 2018	20,510 children aged ≤59 months	Log-binomial and modified Poisson models	National Ecuadorian data, but no detailed IYCF indicators or ML-based prediction.
[38]	Lesotho; MICS 2018	3256 children aged <5 years	Three-level multilevel logistic regression	No ML/XAI approach and limited micronutrient-related variables.
[39]	Malawi; DHS nested cohort	913 children aged <60 months	Matched case–control logistic regression	Context-specific study using non-standardized dietary indicators and no predictive modelling.
[40]	LMICs; systematic review and meta-analysis	451,723 pregnant women and offspring	Systematic review and meta-analysis	Relevant for maternal supplementation, but not focused on IYCF or early-childhood stunting prediction.
[41]	Uganda; UDHS 2016	5485 children aged 6–23 months	Logistic regression; descriptive analysis	Focuses on feeding practices, but does not model anthropometry or stunting.
[42]	Ecuador; LSMS 2006 and 2014	Children aged <5 years with anthropometry	OLS; small area estimation	Ecuador-specific socioeconomic evidence, but no ENDI, detailed IYCF indicators, or ML/XAI approach.

Note: The extended version of this evidence synthesis is provided in Supplementary Table S1.

**Table 2:** Standard chronic undernutrition categories based on height-for-age Z-scores (HAZ).

HAZ Score Range	Interpretation
HAZ ≥−2 SD	Not classified as chronically undernourished
−3≤HAZ<−2 SD	Moderate chronic undernutrition
HAZ <−3 SD	Severe chronic undernutrition

**Table 3:** Core infant feeding practice variables.

Variable	Description
f2_s3_304	Time elapsed between birth and initiation of breastfeeding.
f2_s3_306	Breastfed yesterday (binary).
f2_s3_307_1/2	Exclusive breastfeeding indicators and duration measures.
f2_s3_309, f2_s3_310_a–f	Consumption of liquids other than breast milk (water, formula, cow’s milk, juices, soups).
f2_s3_311	Consumption of solid/semisolid foods in the previous 24 h (binary).
f2_s3_312_a–u	Food-group intake indicators used to compute dietary diversity and complementary feeding patterns.
f2_s3_312_e/g/l/q	Individual dietary diversity indicators identified as highly relevant in SHAP explainability analyses.
f2_s3_313	Frequency of solid or semisolid food intake (ordinal).
f2_s3_308, f2_s3_314–315	Breastfeeding support practices (feeding on demand, bottle use).
f2_s3_317_a	Additional infant feeding behavior indicator included in optimized predictive models.

**Table 4:** Maternal perinatal nutrition variables.

Variable	Description
f2_s4b_408	Folic acid intake during the 3 months preceding pregnancy (binary).
f2_s4b_409_a/b	Folic acid intake during pregnancy and consumption frequency.
f2_s4b_410_a/b	Iron supplementation during pregnancy and frequency.
f2_s4b_411_a/b	Other micronutrient supplementation during pregnancy and frequency.
f2_s4d_444	Birth weight in grams.
f2_s4d_445	Low birthweight indicator (<2500 g).
f2_s4d_441_a/b	Presence and measurement of birth length.

**Table 5:** Contextual, geographic, and survey-design variables.

Variable	Description and Analytic Role
Child age, sex	Child demographic characteristics from the household roster.
quintil, pobreza, nbi_1	Socioeconomic status and household deprivation indicators.
area, prov, region	Geographic residence variables used to represent spatial and contextual variation within the predictive models.
estrato	Survey stratification variable retained as survey-design information; not used to define survey-weighted estimates.
fexp, fexp_lm	Survey expansion factors retained as survey-design variables. These were not applied as sampling weights in descriptive summaries, model training, model evaluation, or SHAP interpretation.
fecha_anio, fecha_mes, fecha_dia	Interview timing variables. When retained in predictive specifications, these were treated as data-collection context variables and not as substantive nutritional or socioeconomic predictors.
Water/sanitation indicators	Household environmental indicators considered as contextual variables when available.

**Table 6:** Missingness summary for major analytic variables.

Domain	Variable	Description	N Missing	Missingness	Handling
Outcome	dcronica_2	Chronic undernutrition status	269	3.1%	Excluded if missing.
Child demographics	grupo_edad_nin	Child age group	0	0.0%	Included as categorical predictor.
Socioeconomic context	quintil	Wealth quintile	51	0.6%	Included as categorical predictor.
Socioeconomic context	pobreza	Poverty status	50	0.6%	Included as categorical predictor.
Geographic context	prov_x	Province	0	0.0%	Included as categorical predictor.
Geographic context	area_x	Area of residence	0	0.0%	Included as categorical predictor.
Infant feeding	f2_s3_306	Breastfed yesterday	634	7.4%	Imputed within the modelling pipeline.
Infant feeding	f2_s3_311	Solid or semisolid food yesterday	634	7.4%	Imputed within the modelling pipeline.
Infant feeding	f2_s3_312_a	Food-group intake indicator	2121	24.6%	Used to derive dietary diversity.
Infant feeding	f2_s3_312_e	Food-group intake indicator	2145	24.9%	Used to derive dietary diversity.
Infant feeding	f2_s3_312_l	Food-group intake indicator	2131	24.7%	Used to derive dietary diversity.
Maternal supplementation	f2_s4b_409_a	Folic acid intake during pregnancy	335	3.9%	Recoded and imputed within the modelling pipeline.
Maternal supplementation	f2_s4b_410_a	Iron intake during pregnancy	335	3.9%	Recoded and imputed within the modelling pipeline.
Maternal supplementation	f2_s4b_411_a	Other micronutrient intake during pregnancy	335	3.9%	Recoded and imputed within the modelling pipeline.
Infant supplementation	f2_s4i_488	Chispaz receipt in last 12 months	262	3.0%	Recoded and imputed within the modelling pipeline.
Birth characteristics	peso_nacer_g	Birth weight in grams, cleaned range 1000–5000 g	7563	87.8%	Not used for record exclusion; imputed when included and interpreted cautiously.

**Table 7:** Birthweight by stunting status among children with valid birthweight and outcome data.

Stunting Status	N	Mean Birthweight (g)	SD	95% CI Lower	95% CI Upper
Non-stunted	849	3174.0	487.5	3141.2	3206.9
Stunted	164	2702.4	548.4	2617.8	2786.9

Note: Birthweight was analysed only among children with valid birthweight values after plausibility filtering to the 1000–5000 g range and non-missing stunting status.

**Table 8:** Unadjusted and adjusted mean birthweight differences by stunting status.

Comparison	Mean Difference (g)	95% CI	*p*-Value	Adjustment
Unadjusted: stunted vs. non-stunted	−471.7	−562.0 to −381.3	<0.001	None
Adjusted: stunted vs. non-stunted	−465.9	−558.8 to −373.1	<0.001	Child age group, area, region, wealth quintile, poverty status, unmet basic needs, maternal folic-acid supplementation, and maternal iron supplementation

Note: Mean differences are expressed as stunted minus non-stunted. Negative values indicate lower mean birthweight among children classified as stunted. Adjusted estimates were obtained using linear regression with robust standard errors.

**Table 9:** Predictive performance of baseline machine-learning models for stunting classification (test set).

Model	Dataset	Accuracy	AUC	Sensitivity	Specificity	Brier	Notes
Logistic Regression	Top-30 features	0.742	0.656	0.371	0.824	0.176	Highest AUC and sensitivity among Top-30 models.
Random Forest	Top-30 features	0.799	0.615	0.029	0.969	0.153	Highest overall accuracy in reduced feature space.
XGBoost	Top-30 features	0.773	0.611	0.086	0.925	0.166	Competitive ensemble performance with moderate discrimination.
CatBoost	Top-30 features	0.773	0.611	0.057	0.931	0.159	Similar performance to XGBoost with strong categorical handling.
Logistic Regression	Full cleaned dataset	0.804	0.576	0.000	0.981	0.186	Highest accuracy but failed to detect positive cases.
Random Forest	Full cleaned dataset	0.804	0.601	0.086	0.962	0.155	Strong overall accuracy with improved calibration.
XGBoost	Full cleaned dataset	0.784	0.640	0.200	0.912	0.158	Improved minority-class detection relative to Random Forest.
CatBoost	Full cleaned dataset	0.794	0.661	0.200	0.925	0.149	Highest AUC and best calibration among full-dataset ensemble models.

**Table 10:** Most relevant optimized machine-learning models for stunting classification.

Dataset	Model	Acc.	AUC	Sens.	Spec.	F1	Key Finding
TOP30_MI	Logistic Regression	0.814	0.645	0.371	0.912	0.419	Highest overall predictive performance after optimization.
FULL_CLEAN	XGBoost	0.737	0.619	0.429	0.805	0.370	Most balanced discrimination between stunted and non-stunted children.
FULL_CLEAN	CatBoost Native	0.577	0.615	0.714	0.547	0.379	Highest sensitivity for minority-class detection.

**Table 11:** Performance of final Optuna-optimized models for stunting classification.

Model	Acc.	AUC	Sens.	Spec.	Prec.	F1	Key Finding
Optimized TOP30 Logistic Regression	0.701	0.615	0.543	0.736	0.311	0.396	Best balance between minority-class detection and overall predictive performance.
Optimized FULL CLEAN CatBoost Native	0.742	0.585	0.371	0.824	0.317	0.342	Higher overall accuracy and improved calibration after optimization.

**Table 12:** Comparison of final ensemble-learning strategies for stunting classification.

Model	Acc.	AUC (95% CI)	Sens.	Spec.	F1	Key Finding
Weighted Blend (70% LR/30% CatBoost)	0.83	0.622 (0.586–0.658)	0.286	0.950	0.377	Highest overall accuracy and specificity.
Weighted Blend (85% LR/15% CatBoost)	0.77	0.624 (0.588–0.660)	0.457	0.836	0.416	Most balanced overall ensemble performance.
Optimized Logistic Regression	0.70	0.615 (0.579–0.651)	0.543	0.736	0.396	Highest sensitivity for minority-class detection.

Note: AUC confidence intervals are approximate 95% confidence intervals calculated using a large-sample normal approximation based on the held-out test-set AUC and test-set class counts.

## Data Availability

The dataset analyzed in this study is publicly available through the Instituto Nacional de Estadística y Censos (INEC) of Ecuador at: https://www.ecuadorencifras.gob.ec/encuesta_nacional_desnutricion_infantil/ (accessed on 17 November 2025). The data can be accessed from the “Resultados” section under “Datos Abiertos”. Further inquiries can be directed to the corresponding author.

## Supplementary Materials

The following supporting information can be downloaded at: https://www.mdpi.com/article/10.3390/nu18142232/s1, Table S1: Extended state of the art on child growth, nutrition, and stunting. Table S2: Definitions, source variables, coding rules, and interpretation of engineered variables used or considered during model development. Table S3: Baseline model specifications used for initial model comparison. Table S4: Hyperparameter search spaces used in Optuna optimization. Table S5: Ranked SHAP feature importance values for the optimized CatBoost model.
