# Supplementary material for: Communication and Health in Chronic Childhood Undernutrition: Explainable Ensemble Learning to Identify Dietary Predictors of Infant Feeding Practices and Maternal Supplementation in Ecuador
Source: Nutrients. 2026 Jul 9;18(14):2232. doi: 10.3390/nu18142232 (PMC13414798; doi:10.3390/nu18142232)
Supplement: Supplementary file 1 [file nutrients-18-02232-s001.zip › nutrients-4393236-supplementary.pdf]

# Supplementary Materials

Communication and Health in Chronic Childhood Undernutrition: Explainable Ensemble Learning to Identify Dietary Predictors of Infant Feeding Practices and Maternal Supplementation in Ecuador

**Table S1.** Extended state of the art on child growth, nutrition, and stunting.

| Article | Data source | Population & age range | Key nutritional exposures | Outcome | Study design | ML use | Main findings | Limitations |
|---|---|---|---|---|---|---|---|---|
| [1] | Three public health centers, West Guji Zone, Ethiopia (May 2023) | 395 mother–child pairs; children <24 months | Maternal nutrition knowledge; IYCF; breastfeeding; complementary feeding; dietary diversity; ANC/PNC; food insecurity; WASH | Stunting (HAZ < -2 SD); latent stunting profiles | Cross-sectional; Bayesian logistic regression (MCMC); Latent Class Analysis | No ML/XAI | 47.3% stunting; three latent profiles; maternal knowledge, education, birth interval $\geq$ 2 years protective; food insecurity, lack of ANC increase risk | Not nationally representative; Ethiopia only; no ML/XAI; limited dietary indicators; no anemia outcome |
| [2] | Child Health Clinic, Shenzhen Social Health Center (2021–2022) | 9,581 children aged 0–36 months | Feeding mode; complementary feeding; parental education; birth order; reading time; interaction time; outdoor time | Stunting; developmental delay (ASQ-3) | Cross-sectional; LASSO + logistic regression; ROC, DCA, calibration; external validation | Yes (LASSO feature selection; nomogram; interpretable ML) | 7.14% stunting; predictors: sex, age, maternal education, birth order, feeding mode, parent–child interaction; AUC = 0.678–0.734 | Uses ASQ-3 (not HAZ); China context; limited IYCF indicators; cross-sectional; not comparable to ENDI |
| [3] | UNICEF–WHO–WB 2023 + simulated dataset | Children 0–5 years (synthetic) | Age; weight; height; calories; hemoglobin | Binary nutritional deficiency | PRISMA review + predictive ML; PCA; clustering; reinforcement learning simulation | Yes: XGBoost; KMeans; PCA; RL | High accuracy in XGBoost; good cluster separation (silhouette 0.75); RL improved simulated hemoglobin | Simulated data; no IYCF; no WHO z-scores; not real anthropometry; no stunting inference |
| [4] | Lulun II longitudinal cohort, rural Cotopaxi, Ecuador | 125 children; mean age 34 months | Ultra-processed food intake (24h FFQ); UPF categories | HAZ; stunting; WHZ; bone age z-score | Longitudinal regression; mediation via BAZ; nutrient profiling | No ML | UPF associated with lower HAZ (beta -0.43); higher stunting OR = 3.07; higher BAZ; savory UPF reduces WHZ | Small sample; not national; limited IYCF; no biomarkers; older toddlers (approx. 3 years) |
| [5] | ENSANUT 2018 (Ecuador) | 20,510 children $\leq$ 59 months | Food insecurity (FIES); water insecurity (HWI); joint HFI–HWI | Stunting; diarrhea; respiratory illness | National cross-sectional; log-binomial and modified Poisson; interaction HFI x HWI | No | HFI increases diarrhea (PR 1.39) and RI (PR 1.34); HWI increases RI; joint HFI–HWI increases diarrhea and RI; no stunting association | No IYCF; no diet details; cross-sectional; stunting not associated despite biological plausibility |

**Table S1.** *Cont.*

| Article | Data source | Population & age range | Key nutritional exposures | Outcome | Study design | ML use | Main findings | Limitations |
|---|---|---|---|---|---|---|---|---|
| [6] | MICS 2018 (national, multistage) | 3,256 children <5 years | MAD; birthweight; infections; maternal education; poverty; WASH | Stunting (HAZ < -2 SD) | Three-level multilevel logistic regression | No | Stunting associated with poor MAD, low BW, infection, poverty, low maternal education, unsafe water and sanitation; strong community-level effects | No detailed IYCF; no micronutrients; results not comparable to ENSANUT; no ML |
| [7] | DHS 2010 nested cohort (Malawi) | 913 children <60 months (matched pairs) | EBF; complementary foods; unhealthy foods; maternal MDD-W; HFIAS; polygamy; infections | Stunting (HAZ < -2 SD) | Matched case–control; adjusted logistic regression | No | Stunting associated with male sex, older age, polygamy, low maternal education, infections, food insecurity, poor maternal diet | Non-standard IYCF; context-specific; heterogeneous variables; no predictive models |
| [8] | 72 studies in LMIC (RCTs + quasi-experimental) | 451,723 pregnant women + offspring | Micronutrient supplementation: IFA, iron, zinc, vitamin A/D, calcium, MMN, LNS | LBW; SGA; preterm; stillbirth; diarrhea | Systematic review + meta-analysis | No | MMN reduces LBW (RR 0.85), SGA (RR 0.93), stillbirth (RR 0.91); IFA reduces anemia; limited data on child anthropometry | Not focused on IYCF or stunting 0–24m; limited infant height data |
| [9] | UDHS 2016 (Uganda) | 5,485 children 6–23 months | IYCF indicators: MDD; MMF; MAD | No anthropometry | Logistic regression; descriptive stratified by age | No | MDD low (26–34%); MMF adequate; illness, vaccination, wealth increase IYCF; empowerment unrelated | No HAZ; no stunting modeled; CF focus only |
| [10] | LSMS 2006 & 2014 (Ecuador) | Children <5 years (n=6003; n=11,473) with anthropometry | Relative deprivation; own/relative consumption; breastfeeding; parental height/weight | Stunting (WHO HAZ) | OLS; province/county/parish models; SAE for Gini | No | Higher relative deprivation decreases HAZ; breastfeeding mitigates adverse effects; inequality non-significant | No ENSANUT; no IYCF; no morbidity; limited food security variables |

**Table S2.** Definitions, source variables, coding rules, and interpretation of engineered variables used or considered during model development.

| Engineered variable | Source variable(s) | Formula / coding rule | Interpretation |
|---|---|---|---|
| `dietary_diversity_scr` | `f2_s3_312_a`–`f2_s3_312_t` | Each food-group item was recoded as 1 for reported consumption in the previous 24 hours and 0 otherwise. The score was calculated as $\sum_{j=1}^{20} I(\text{food group}_j = 1)$. | Number of food-group indicators reported in the previous 24 hours. |
| `dietary_diversity_adqt` | `dietary_diversity_scr` | Coded as 1 if `dietary_diversity_scr` $\geq$ 4 and 0 otherwise. | Binary indicator of dietary diversity adequacy using the threshold applied in the analysis. |
| `bf_months` | `f2_s4f_467_b` | Converted to numeric months; non-numeric responses were treated as missing. | Reported breastfeeding duration in months. |
| `continued_bf_12m` | `bf_months` | Coded as 1 if `bf_months` $\geq$ 12 and 0 otherwise, when breastfeeding duration was available. | Indicator of breastfeeding continuation at or beyond 12 months. |
| `bf_yesterday` | `f2_s3_306` | Coded as 1 for affirmative responses and 0 otherwise. | Reported breastfeeding on the previous day. |
| `solid_yesterday` | `f2_s3_311` | Coded as 1 for affirmative responses and 0 otherwise. | Reported solid or semisolid food intake on the previous day. |
| `chispaz_bin` | `f2_s4i_488` | Coded as 1 for affirmative responses and 0 otherwise. | Reported Chispaz receipt/use according to the ENDI item. |
| `af_preg_bin` | `f2_s4b_409_a` | Coded as 1 for affirmative responses and 0 otherwise. | Reported maternal folic-acid intake during pregnancy. |
| `iron_preg_bin` | `f2_s4b_410_a` | Coded as 1 for affirmative responses and 0 otherwise. | Reported maternal iron intake during pregnancy. |
| `other_mn_preg_bin` | `f2_s4b_411_a` | Coded as 1 for affirmative responses and 0 otherwise. | Reported maternal intake of other micronutrients during pregnancy. |
| `peso_nacer_g` | `f2_s4d_444` | Converted to numeric grams. Values outside 1000–5000 g were recoded as missing for this variable only. | Reported birthweight in grams after plausibility filtering. |
| `lbw` | `peso_nacer_g` or `f2_s4d_445` | Coded as 1 for birthweight $<$2500 g and 0 for birthweight $\geq$2500 g when valid birthweight information was available. | Low-birthweight indicator. |

Note: Missingness for major source variables is reported in Table 6. This supplementary table focuses on coding rules and transformations used to construct engineered variables from the original ENDI questionnaire items. 5

**Table S3.** Baseline model specifications used for initial model comparison.

| Model | Preprocessing | Baseline specification |
|---|---|---|
| Logistic Regression | Median imputation and standardization for numerical variables; most-frequent imputation and one-hot encoding for categorical variables. | `max_iter=2000`, `solver=lbfgs`, `class_weight=balanced`, `random_state=42`. |
| Random Forest | Median imputation for numerical variables; most-frequent imputation and one-hot encoding for categorical variables. | `n_estimators=400`, `min_samples_leaf=5`, `class_weight=balanced`, `random_state=42`. |
| XGBoost | Median imputation and standardization for numerical variables; most-frequent imputation and one-hot encoding for categorical variables. | `n_estimators=400`, `max_depth=4`, `learning_rate=0.05`, `subsample=0.8`, `colsample_bytree=0.8`, `eval_metric=logloss`, `scale_pos_weight` calculated from the training-set class ratio, `random_state=42`. |
| CatBoost | Native handling of categorical variables; categorical predictors were passed directly to CatBoost. | `iterations=2000`, `learning_rate=0.01`, `depth=5`, `l2_leaf_reg=6`, `loss_function=Logloss`, `eval_metric=AUC`, `auto_class_weights=Balanced`, `random_seed=42`. |

**Table S4.** Hyperparameter search spaces used in Optuna optimization.

| Model | Hyperparameter | Search space |
|---|---|---|
| Logistic Regression | `C` | Log-uniform range from 0.001 to 20.0. |
| Logistic Regression | `penalty` | Categorical choice between `l1` and `l2`. |
| Logistic Regression | `class_weight` | Categorical choice between `balanced` and no class weighting. |
| Logistic Regression | `solver` | Fixed as `liblinear`. |
| CatBoost Native | `iterations` | Integer range from 800 to 5000. |
| CatBoost Native | `learning_rate` | Log-uniform range from 0.003 to 0.05. |
| CatBoost Native | `depth` | Integer range from 3 to 7. |
| CatBoost Native | `l2_leaf_reg` | Log-uniform range from 3.0 to 80.0. |
| CatBoost Native | `random_strength` | Uniform range from 0.5 to 10.0. |
| CatBoost Native | `bagging_temperature` | Uniform range from 0.0 to 10.0. |
| CatBoost Native | `border_count` | Categorical choice among 32, 64, 128, and 254. |
| CatBoost Native | `auto_class_weights` | Categorical choice between `Balanced` and `SqrtBalanced`. |
| CatBoost Native | `loss_function` | Fixed as `Logloss`. |
| CatBoost Native | `eval_metric` | Fixed as `F1`. |
| CatBoost Native | `bootstrap_type` | Fixed as `Bayesian`. |
| CatBoost Native | Early stopping | Stopped after 100 rounds without validation improvement during cross-validation. |

**Table S5.** Ranked SHAP feature importance values for the optimized CatBoost model.

| Rank | Feature | Domain | Mean absolute SHAP | Interpretation |
|---|---|---|---|---|
| 1 | `prov_x` | Geographic context | 0.195095 | Province contributed the largest average SHAP magnitude to model prediction. |
| 2 | `area_x` | Geographic context | 0.166461 | Area of residence contributed strongly to the model's predictive structure. |
| 3 | `f2_s3_307_2` | Infant feeding | 0.151483 | Feeding-related variable contributing to predicted stunting classification. |
| 4 | `region_y` | Geographic context | 0.142035 | Regional context variable contributing to model prediction. |
| 5 | `area_y` | Geographic context | 0.115084 | Area-related variable contributing to model prediction. |
| 6 | `f2_s3_304` | Infant feeding | 0.113043 | Feeding-related variable contributing to predicted classification. |
| 7 | `f2_s3_312_g` | Infant feeding | 0.109984 | Food-group intake indicator contributing to model prediction. |
| 8 | `f2_s3_313` | Infant feeding | 0.099492 | Feeding-related variable contributing to model prediction. |
| 9 | `fecha_mes_x` | Survey/design | 0.097153 | Interview timing variable retained as a non-substantive model covariate. |
| 10 | `fexp` | Survey/design | 0.091816 | Expansion factor retained as a non-substantive model covariate, not applied as a sampling weight. |
| 11 | `region_x` | Geographic context | 0.084318 | Regional context variable contributing to model prediction. |
| 12 | `f2_s4b_408` | Maternal supplementation | 0.083979 | Maternal folic-acid related variable contributing to model prediction. |
| 13 | `f2_s3_312_l` | Infant feeding | 0.080819 | Food-group intake indicator contributing to model prediction. |
| 14 | `nbi_1` | Socioeconomic context | 0.078437 | Unmet basic needs contributed to model prediction. |
| 15 | `f2_s3_310_b_2` | Infant feeding | 0.074282 | Feeding-frequency related variable contributing to model prediction. |
| 16 | `f2_s3_310_a_1` | Infant feeding | 0.071413 | Feeding-frequency related variable contributing to model prediction. |
| 17 | `f2_s3_312_q` | Infant feeding | 0.063280 | Food-group intake indicator contributing to model prediction. |
| 18 | `fexp_lm` | Survey/design | 0.060660 | Module-specific expansion factor retained as a non-substantive covariate. |
| 19 | `fecha_dia_x` | Survey/design | 0.058025 | Interview timing variable retained as a non-substantive model covariate. |
| 20 | `f2_s3_312_e` | Infant feeding | 0.057763 | Food-group intake indicator contributing to model prediction. |

Note: SHAP importance values are reported as mean absolute SHAP values. They quantify the average magnitude of each predictor's contribution to model predictions and do not indicate causal, epidemiological, or directional associations. Survey/design variables were retained in the ranked output for transparency but were not interpreted as substantive predictors.

1. Fikrie, A.; Adula, B.; Beka, J.; Hailu, D.; Kitabo, C.A.; Spigt, M. Analysis of Determinants of Stunting and Identifications of Stunting Risk Profiles Among Under 2-Year-Old Children in Ethiopia. A Latent Class Analysis. *Health Services Research and Managerial Epidemiology* **2024**, *11*, 23333928241271921.
2. Xiong, Y.; Hu, X.; Cao, J.; Shang, L.; Niu, B. A predictive model for stunting among children under the age of three. *Frontiers in Pediatrics* **2024**, *12*, 1441714.
3. Davalos Carrera, J.I.; Cortez Paredes, K.M. Modelos de machine learning para la detección temprana de deficiencias nutricionales en la infancia. B.S. thesis, 2025.
4. Gyimah, E.A.; Nicholas, J.L.; Waters, W.F.; Gallegos-Riofrío, C.A.; Chapnick, M.; Blackmore, I.; Douglas, K.E.; Iannotti, L.L. Ultra-processed foods in a rural Ecuadorian community: associations with child anthropometry and bone maturation. *British Journal of Nutrition* **2023**, *130*, 1609–1624.
5. Chakraborty, R.; Armijos, R.X.; Beidelman, E.T.; Rosenberg, M.; Margaret Weigel, M. Household food and water insecurity and its association with diarrhoea, respiratory illness, and stunting in Ecuadorian children under 5 years. *Maternal & Child Nutrition* **2024**, *20*, e13683.
6. Leseba, N.; Vermaak, K.; Makatjane, T.; Lebuso, M. A multilevel analysis of factors associated with stunting among children under five years in Lesotho: a study of the lesotho multiple cluster indicator survey 2018. *Journal of Health, Population and Nutrition* **2025**, *44*, 168.
7. Fungo, R.; Zulu, R.; Munthali, J.; Mutua, M.; Birachi, E. Factors associated with stunting among children aged below 60 months from rural Malawi: A matched case-control study. *African Journal of Food, Agriculture, Nutrition and Development* **2023**, *23*, 25024–25051.
8. Keats, E.C.; Oh, C.; Chau, T.; Khalifa, D.S.; Imdad, A.; Bhutta, Z.A. Effects of vitamin and mineral supplementation during pregnancy on maternal, birth, child health and development outcomes in low-and middle-income countries: A systematic review. *Campbell Systematic Reviews* **2021**, *17*, e1127.
9. Scarpa, G.; Berrang-Ford, L.; Galazoula, M.; Kakwangire, P.; Namanya, D.B.; Tushemerirwe, F.; Ahumuza, L.; Cade, J.E. Identifying predictors for minimum dietary diversity and minimum meal frequency in children aged 6–23 months in Uganda. *Nutrients* **2022**, *14*, 5208.
10. Larrea, A.; Ramos, X. Relative deprivation and malnutrition in Ecuador **2023**.
